# The Mitochondrial Phosphate Transporters Modulate Plant Responses to Salt Stress via Affecting ATP and Gibberellin Metabolism in *Arabidopsis thaliana*

**DOI:** 10.1371/journal.pone.0043530

**Published:** 2012-08-24

**Authors:** Wei Zhu, Qing Miao, Dan Sun, Guodong Yang, Changai Wu, Jinguang Huang, Chengchao Zheng

**Affiliations:** State Key Laboratory of Crop Biology, College of Life Sciences, Shandong Agricultural University, Taian, Shandong, People’s Republic of China; UMass, United States of America

## Abstract

The mitochondrial phosphate transporter (MPT) plays crucial roles in ATP production in plant cells. Three *MPT* genes have been identified in *Arabidopsis thaliana*. Here we report that the mRNA accumulations of *AtMPTs* were up-regulated by high salinity stress in *A. thaliana* seedlings. And the transgenic lines overexpressing *AtMPTs* displayed increased sensitivity to salt stress compared with the wild-type plants during seed germination and seedling establishment stages. ATP content and energy charge was higher in overexpressing plants than those in wild-type *A. thaliana* under salt stress. Accordingly, the salt-sensitive phenotype of overexpressing plants was recovered after the exogenous application of atractyloside due to the change of ATP content. Interestingly, Genevestigator survey and qRT-PCR analysis indicated a large number of genes, including those related to gibberellin synthesis could be regulated by the energy availability change under stress conditions in *A. thaliana*. Moreover, the exogenous application of uniconazole to overexpressing lines showed that gibberellin homeostasis was disturbed in the overexpressors. Our studies reveal a possible link between the ATP content mediated by AtMPTs and gibberellin metabolism in responses to high salinity stress in *A. thaliana*.

## Introduction

High salinity is an important abiotic stress that is commonly encountered by plants growing in their native environments [1]–[3]. To survive this challenge, plants have developed elaborate mechanisms to perceive external signals and manifest adaptive responses with proper physiological changes [4]. Mitochondria can integrate numerous metabolic pathways that are important in adaptive responses to extreme environmental conditions, such as respiration and oxidative phosphorylation, the control of redox balance, and the metabolism of proline and ascorbate [5], [6]. Among these processes, respiration is the core process of mitochondrial metabolism; at the same time, a large amount of free energy is released and used for adenosine triphosphate (ATP) production by oxidative phosphorylation [7], [8]. Previous studies demonstrated that many physiological and molecular responses in plant cells were associated with energy state which could be reflected by energy charge [9]. Energy charge modulates the activity of various metabolic events related to energy utilization and regeneration. When the energy charge is greater than 0.5, the activities of ATP-utilizing systems increase, and conversely, ATP-regeneration systems are dominant [10], [11]. Therefore, elucidating the relationship between energy state and stress tolerance is an important challenge. Recently, increasing numbers of reports have indicated that transcriptome reprogramming in energy and stress signaling is partly regulated by the evolutionarily conserved energy sensor protein kinases (SNF1) in yeast, and AMP-activated protein kinase (AMPK) in mammals [12]. However, the intersection points between the energy and stress pathway are largely under explored in plants. The only emerging view is that two *A. thaliana* protein kinases, KIN10 and KIN11, are identified as central regulators of the transcriptome in response to energy-deficiency and multiple types of stress signals, providing new insight into the molecular mechanisms underlying energy and stress signaling [13].

It has been reported that uptake of orthophosphate (Pi) into the mitochondrial matrix is essential for the oxidative phosphorylation of ADP to ATP [14]. The mitochondrial phosphate transporter/carrier (MPT/PiC), located in the mitochondrial inner membrane, catalyzes the phosphate (H_2_PO_4_−)/proton symport, the phosphate/hydroxyl ion antiport, and the exchange of mitochondrial matrix with cytosolic phosphate [15]–[17]. In mammals, the two isoforms of PiC A and B, differing in the sequence near the N terminus, arise from alternative splicing of a primary transcript of the *PiC* gene. Isoform A is present in high amounts in heart, skeletal muscle, and diaphragm tissues to match the higher energy demands, whereas isoform B is present in all tissues to provide basic energy requirements [18], [19]. Meanwhile, the deficiency of the mitochondrial phosphate carrier SLC25A3 has been proved to lead to a disorder in the synthesis of ATP [20]. In yeast, a PiC form encoded by the *MIR1* gene has been identified [17], [21]. And PIC in yeast been reported to function not in monomeric form and its dimers are stable both in the solubilized state and after membrane insertion [22].

In plants, increasing numbers of reports have indicated that plant MPT may play similarly important roles as they do in animals and yeast. First, an ozone-inducible gene was reported to encode a putative MPT in birch [15]. Afterwards, the *MPT* genes have been isolated and characterized in various plant species, including soybean, maize, rice, *Arabidopsis* and *Lotus japonicus* [16], [23]. The phosphate transport activity of the soybean and *L. japonicus* MPT protein was confirmed by reconstitution of proteoliposomes and phosphate transport assay [16], [23]. The ectopic expression of *A. thaliana* mitochondrial phosphate carriers genes, *AT5* (*At5g14040*) and *AT3* (*At3g48850*), can complement a yeast mutant lacking the endogenous PiC and restore phosphate transport activity [17]. In addition, MPT might play an important role during the release of bud dormancy in *Paeonia suffruticosa* [24]. To date, knowledge on the molecular mechanisms of MPT-mediated biological functions in plants is still limited.

The phytohormone gibberellin (GA) regulates many aspects of plant growth and developmental processes [25], as well as responses to the environmental stimuli [26]–[29]. Previous studies have clearly demonstrated that the amount of total bioactive gibberellin can be affected by both the rate of their synthesis and the conversion to inactive forms, two processes catalyzed by three categories of dioxygenases, which are encoded by *GA20ox*, *GA3ox*, and *GA2ox* genes in *A. thaliana* [30], [31]. In addition, the endogenous gibberellin levels are regulated by environmental stimuli as well [32], [33]. Recently, gibberellin was shown to play an important role in plant salt stress tolerance through regulating DELLA class transcription factors in *A. thaliana*. The reduced gibberellins in *A. thaliana* can increase DELLA levels, which contributes to growth retardation under salt stress [29]. The overexpression of gibberellin responsive genes and exogenous application of gibberellins are able to counteract the inhibitory effects of salt, oxidative, and heat stresses in seed germination and seedling growth via modulation of salicylic acid biosynthesis [34]. Taken together, these data provide a novel insight into the involvement of plant hormones in stress tolerance. However, the relationship between gibberellin levels and mitochondrial phosphate transporters under salt stress is still unclear.

In this paper we report that the expression level of *A. thaliana MPT* genes is significantly induced under high salinity conditions, and the sensitivity of the *AtMPT* overexpressors to salt stress might be due to an increase in ATP content. Furthermore, gibberellins might be involved in AtMPT-related early responses to high salinity stress in *A. thaliana*. Our results provide evidence of interrelatedness of ATP, gibberellins, and salt stress response in plants for the first time.

## Results

### The Characteristic of AtMPTs

To understand the biological functions of MPT in plants, a total of three *MPT* genes have been identified in the *A. thaliana* genome, which we designate here as *AtMPT1* (*AT2G17270*), *AtMPT2* (*AT3G48850*), and *AtMPT3* (*AT5G14040*). In other reports, they are named *AT2*, *AT3, AT5* [17] and *PHT3; 3*, *PHT3; 2*, *PHT3; 1* [35]. The open reading frames of *AtMPT*1, *AtMPT*2, and *AtMPT*3 encode proteins of 309, 363 and 375 amino acids, with predicted molecular masses of 34.3, 39.0 and 40.1 kD, respectively. By amino acid sequence alignment, 65.87% sequence identity is observed between AtMPT2 and AtMPT3. In contrast, AtMPT1 shows only 41.87% sequence identity with AtMPT2 and 42.13% sequence similarity with AtMPT3 ().

**Figure 1:**
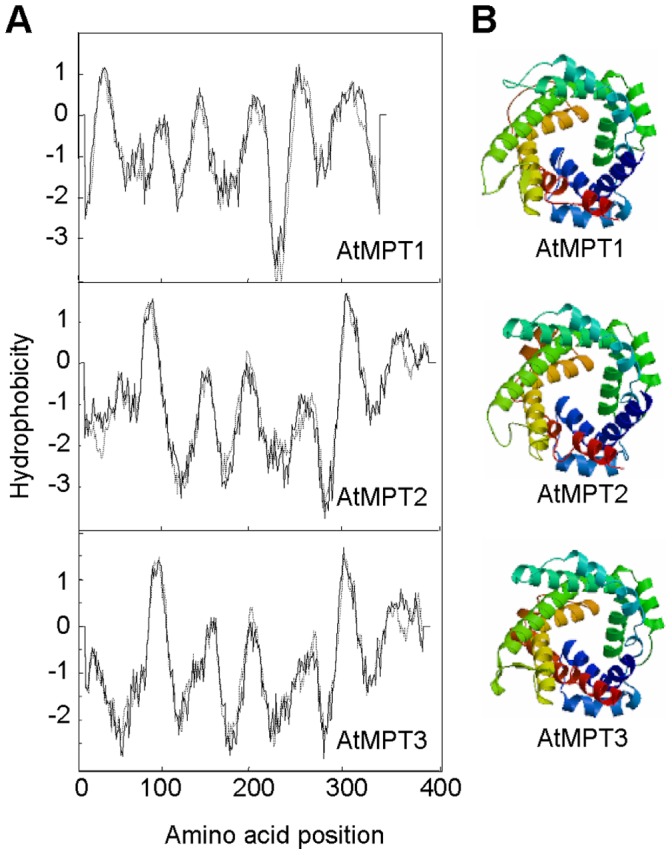
AtMPT structure prediction. (**A**) Transmembrane topology prediction for AtMPT1, AtMPT2, and AtMPT3. Hydropathy values were analyzed by TMPred prediction (http://www.ch.embnet.org/software/TMPRED_form.html). (**B**) Three- dimensional structures were predicted using their pdb coordinates and the Swiss-Model program (http://swissmodel.expasy.org).

Hydropathy prediction (http://www.ch.embnet.org/software/TMPRED_form.html) and SWISS-MODEL (http://swissmodel.expasy.org) analysis identified that the AtMPTs have three tandem domains consisting of about 100 amino acids, which are made up of two transmembrane α-helices separated by a hydrophilic extra-membrane loop (Figure 1A, B). These domains, which are essential for mitochondrial targeting, are also conserved in all analyzed mitochondrial transporter proteins (Figure S1). However, the key residues for phosphate transport function of mammalian and yeast MPTs display inconsistent identities in *A. thaliana*, implying an activity difference or functional diversity (Figure S1). Five residues (His-32, Asp-39, Glu-126, Glu-137 and Asp-236) are shown critical for transport activity in yeast [36]. All of them are identical in AtMPT2 and AtMPT3, whereas in AtMPT1 Glu-126 occurs instead of Gln-122. Another essential residue Cys-42 for bovine MPTs activity [37] is also conserved in AtMPT2 and ATMPT3 but not in AtMPT1. Overall, these data suggest that the AtMPT proteins have the features of a mitochondrial phosphate transporter.

### The Distinct Temporal and Spatial Expression of the Three *MPT* Genes in *A. thaliana*

**Figure 2:**
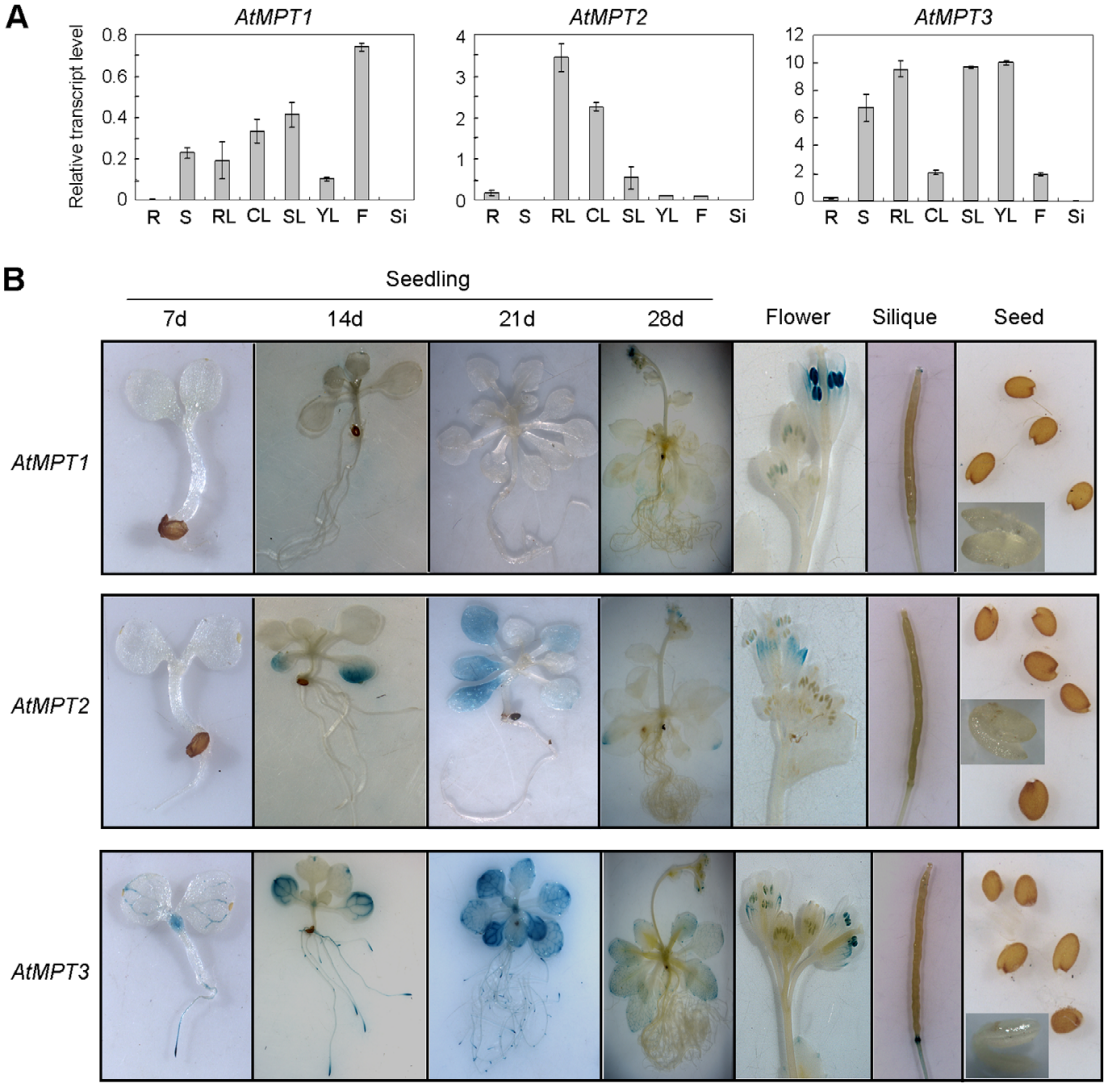
Expression patterns of the *AtMPT*s. (**A**) Tissue-specific RNA expression. Total RNA was isolated from roots (R), primary shoot (S), rosette leaf (RL), cauline leaf (CL), flower (F), and siliques (Si) of 28-day-old wild-type (Col-0) plants and young rosette leaf (YL) and senescent rosette leaf (SL) of 14-day-old wild-type plants. *AtMPT* transcript levels were analyzed by quantitative real-time RT-PCR using gene-specific PCR primers. The transcript levels were normalized to that of elongation factor 1-a (EF1-α). Values are means±SEM of three replicates. (**B**) Histochemical analysis of expression patterns of the *AtMPTs*. Glucuronidase (GUS) activities in transgenic *A. thaliana* carrying the *AtMPT1*, *AtMPT2,* or *AtMPT3 promoter::GUS* constructs were analyzed by staining with X-gluc. Images were shown of flowers, siliques, seeds (embryos) and *A. thaliana* seedlings at different developmental stages.

To assess the role of individual MPTs in *A. thaliana*, we examined the expression of *AtMPT1*, *AtMPT2*, and *AtMPT3* using quantitative real-time reverse transcription-PCR (qRT-PCR) and *promoter::β-glucuronidase* (*GUS*) fusions. Our qRT-PCR data demonstrated that the transcripts of the *AtMPTs* could be detected in all tissues except the siliques (Figure 2A). *AtMPT1* was expressed at a lower level than the other two *AtMPT*s. And the levels of *AtMPT2* mRNA were high in rosette leaves, whereas *AtMPT3* strongly expressed in leaves but weakly in the roots and flowers. For a more detailed analysis of the expression patterns, promoter fragments covering about 1 kb upstream of the translational start site were cloned for *AtMPT1*, *AtMPT2* and *AtMPT3* fused to *GUS*, and transformed into *A. thaliana* plants (Columbia ecotype). As shown in Figure 2B, GUS-staining of the transgenic plants revealed that the expression of *AtMPT1* was strong in the stamens of flowers. *AtMPT2* showed particularly high expression in senescent leaves. Strong expression of *AtMPT3::GUS* was detected especially in vascular tissues, but also in roots, rosette leaves, and the meristems of young seedlings. Generally, the above GUS-staining results were in accordance with the qRT-PCR detection, suggesting the expression level of *AtMPT3* was the highest among the three *AtMPT* genes. However, no staining of *AtMPT1::GUS* in tissues except stamen was observed, which might be due to the low abundance of *AtMPT1* transcripts, and weak GUS expression in some sepals of *AtMPT2* transgenic plants was detected. Overall, these data indicate that the expression patterns of three *AtMPT* genes are different from each other, implying they play specific roles in various organs or developmental stages.

### AtMPTs Involved in Plant Response to High Salinity Stress

The lines overexpressing *AtMPTs* under control of the cauliflower mosaic virus 35S promoter (OEMPTs) were used to investigate the functions of AtMPTs. Seven independent transgenic lines overexpressing *AtMPT1* (L1–7), six transgenic lines overexpressing *AtMPT2* (L8–13), and four transgenic lines overexpressing *AtMPT3* (L14–17) were generated (Data not shown). Among these transgenic plants, T3 homozygous L5 (OEMPT1), L8 (OEMPT2), and L14 (OEMPT3), which showed the highest expression levels of *AtMPT1*, *AtMPT2,* and *AtMPT3,* respectively, were selected for further analysis.

**Figure 3:**
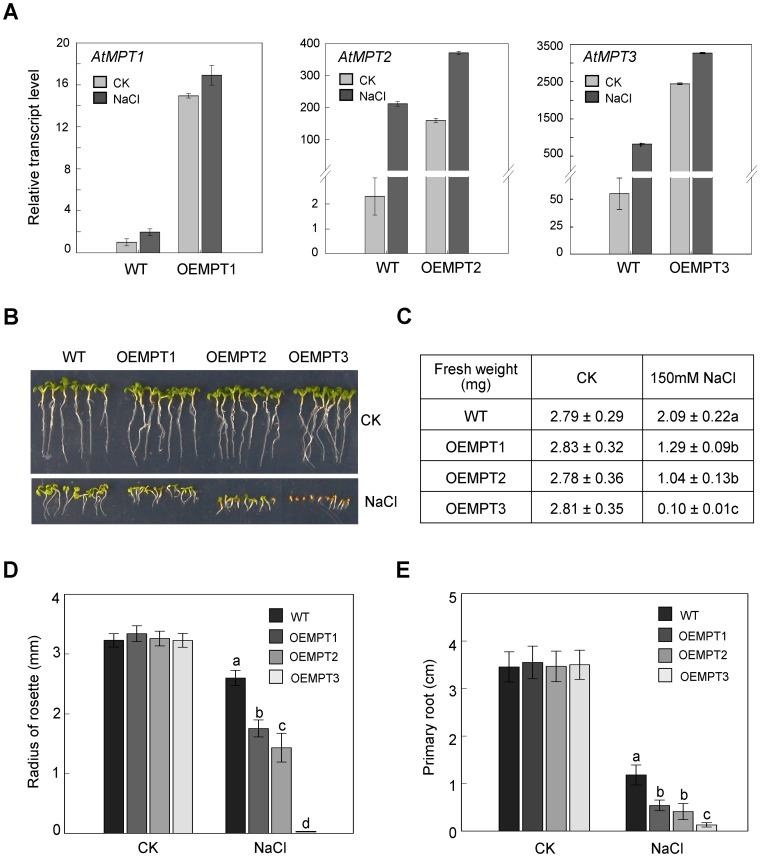
The roles of AtMPT in salt stress response. (**A**) qRT-PCR analysis of *AtMPT* transcripts in wild-type plants and transgenic plants overexpressing *AtMPTs* under control and salt stress conditions. Ten-day-old seedlings grown on 1/2 MS agar media were transferred onto filter paper soaked with water and 150 mM NaCl for 24 h. The transcript level in the wild-type sample for *AtMPT1* under control conditions was set to 1, and the other levels were calculated relative to the corresponding value. Values are means±SEM of three replicates. (**B**) Photographs of wild-type and overexpression lines were taken 10 days after germination on 1/2 MS agar media and 1/2 MS agar media with 150 mM NaCl. (**C**) Mean fresh weights (FW) of 10-day-old plants. (**D**) Mean radius of rosettes of 10-day-old plants. The overexpression lines for *AtMPT3* under salt stress were damaged and had no normal rosette leaves to measure. Error bars represent±SEM. (**E**) Mean primary root lengths of 10-day-old plants. Error bars represent±SEM. Measurements shown in Figure C–E were made 10 days post-germination under control and high salinity stress conditions (n = 30). Samples with different letters are significantly different: P<0.01. OEMPTs, the *AtMPT* overexpressors.

To explore the functions of AtMPTs in *A. thaliana* response to salt stress, we examined the effects of high salinity stress on *AtMPT* transcription. As shown in Figure 3A, in wild-type plants after 150 mM NaCl treatment, the expression of *AtMPT1*, *AtMPT2,* and *AtMPT3* increased approximately 2-, 100-, and 12-fold, respectively. And in *AtMPT* overexpressors, the transcript levels of *AtMPTs* appeared higher compared to wild-type plants in response to salt treatment (Figure 3A). Furthermore, both the seed germination and seedling establishment of the overexpression lines were clearly inhibited when they grew vertically on 1/2 MS agar media with 150 mM NaCl (Figure 3B). Especially, biomass accumulation, leaf expansion and primary root length were strongly affected compared to that of the wild-type plants (Figure 3C, D and E). To confirm this, two additional independent overexpression lines of the three *AtMPT*s also showed the same sensitive phenotype (Figure S2). And the sensitivities of the overexpressors were similar to wild-type plants when they were treated with 285 mM mannitol, which gives rise to a similar osmotic stress as 150 mM NaCl, suggesting that the increased salt stress sensitivity was mainly caused by salt ion toxicity (Figure S3). To determine whether the phenotype of the overexpressors was regulated by altered calcium balance, we increased the calcium level in the growth medium (∼1∶20 ratio to Na^+^). The additional calcium scarcely restored sensitive phenotype, implying that calcium level has little direct influence on the phenotype of the overexpressors (Figure S4). Altogether, these observations indicate that AtMPTs regulate *A. thaliana* salt stress responses.

### ATP Plays an Essential Role in AtMPT-mediated Early Responses to Salt Stress

**Figure 4:**
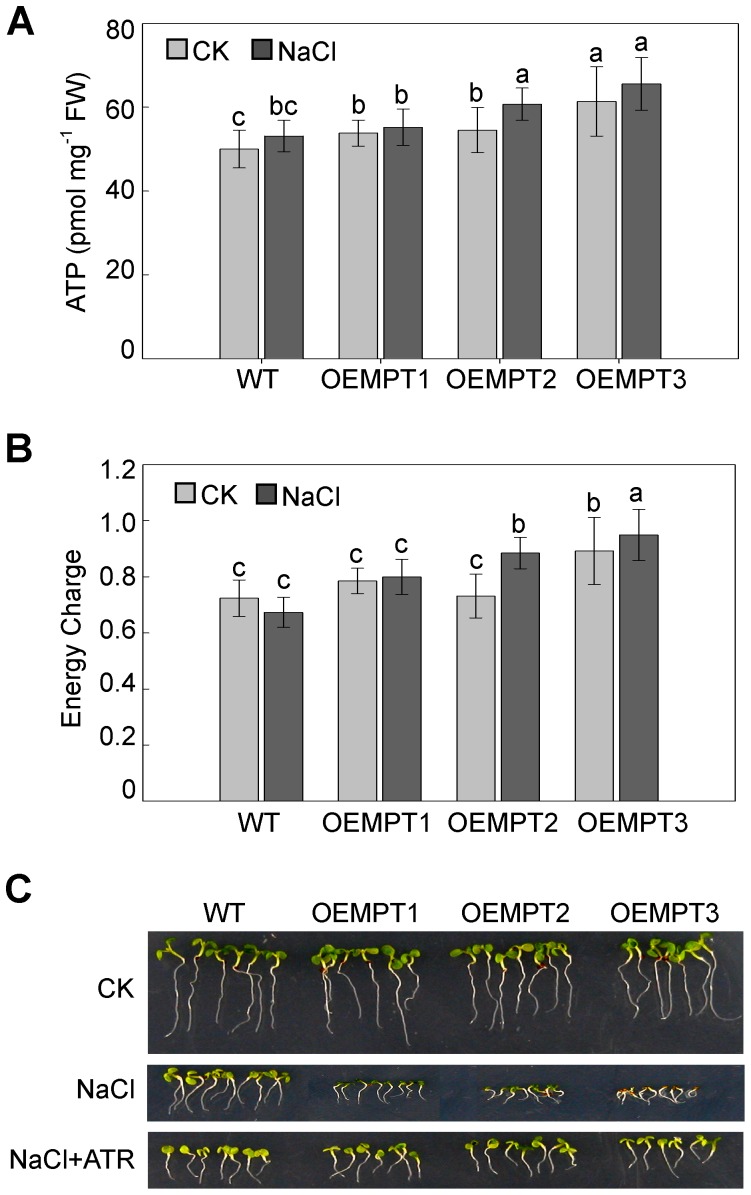
AtMPT mediates salt stress tolerance through an ATP-dependent pathway. (**A**) ATP content in 10-day-old wild-type plants and the overexpression lines under control and 150 mM NaCl conditions. Values represent the average of three replicates±SEM. (**B**) Energy charge of 10-day-old wild-type plants and overexpression lines under control and salt stress. Values represent the average of three replicates±SEM. (**C**) Wild type and overexpressing seedlings germinated and grown under control and 150 mM NaCl together with 10 µM atractyloside. The pictures were taken 10 days post-germination. Samples with different letters are significantly different: P<0.05 (a and b, b and c). OEMPTs, the *AtMPT* overexpressors.

To explore the reasons behind the enhanced sensitivity to salt stress of *AtMPT* overexpressors, we measured ATP content and energy charge in these plants under control and salt stress conditions. A slight increase of ATP content in the overexpressors compared with wild-type plants was detected under both control and salt stress (Figure 4A). The energy charge was increased approximately 10% in the overexpressors compared to wild-type plants under control and salt stress (Figure 4B). These results indicate that energy states might be correlated with the expression levels of AtMPTs.

To verify whether the increase of ATP content lead to enhanced salt sensitivity of the overexpressors, we performed complementation assays with atractyloside, an ATP/ADP carrier inhibitor. The seeds of the overexpression lines were treated with the same NaCl stress together with 10 µM atractyloside. As shown in Figure 4C, salt sensitivities of the overexpressors were almost identical to wild-type plants during seed germination and seedling establishment when the exchange of ATP was blocked. These results confirm that the changes of available energy lead to the high salinity sensitive phenotypes of *AtMPT* overexpressors.

### The Expression of Gibberellin Metabolism Genes were Regulated by AtMPTs

**Figure 5:**
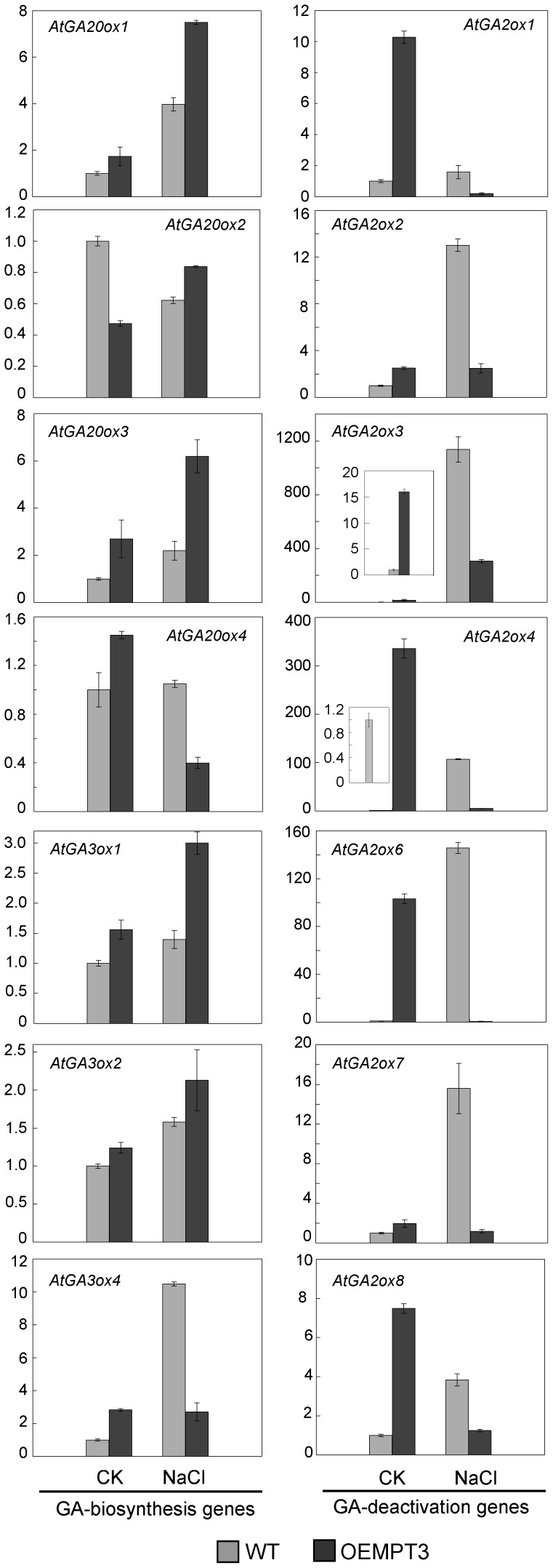
Expression levels of the genes involved in gibberellin metabolism. Expression levels of *GA20ox*, *GA3ox*, and *GA2ox* genes in wild-type and the *AtMPT3* overexpression lines under control and salt stress. The mRNA levels of these genes were normalized against transcript levels of EF1-α. The transcripts of *GA20ox5*, *GA3ox3* and *GA2ox5* were not detected. Transcript level in the wild-type sample for each gene under control conditions was set to 1, and the other levels were calculated relative to the corresponding value. Values are means of three replicates with±SEM. OEMPTs, the *AtMPT* overexpressors.

To figure out the possible consequences resulting from the changes of ATP levels during salt stress, we surveyed the public transcriptome databases using the genome tool Genevestigator. A total of 47 salt-reduced genes that are activated by dark (a decrease in energy) or light (an energy accumulation) and 45 salt-induced genes that are repressed by dark or light were identified (Figure S5). And these genes are related to energy metabolism and salt stress response. To gain insight into their biological processes, we analyzed these genes that were significantly differentially expressed (>2-fold) for gene ontology (GO) (http://omicslab.genetics.ac.cn/GOEAST/). 32 genes encoding pivotal transcription factors or involving metabolism of several hormones were identified and then assayed by qRT-PCR (Table S2). Interestingly, the expression of gibberellin metabolism genes changed appreciably in the *AtMPT3* overexpression lines compared with wild-type plants in the salt stress condition. The salt-induced mRNA levels of the gibberellin synthesis genes *GA20ox1/2/3* and *GA3ox1/2/4* in *AtMPT3* overexpressors were slightly higher than that in wild-type plants, whereas the induced transcriptional levels of most gibberellin deactivation genes *GA2oxs* were lower than that in wild-type plants (Figure 5). These results reveal gibberellin synthesis pathways are over-activated by high AtMPT3 expression levels under salt stress.

### Gibberellin Affects AtMPT-mediated Early Responses to Salt Stress in *A. thaliana*

**Figure 6:**
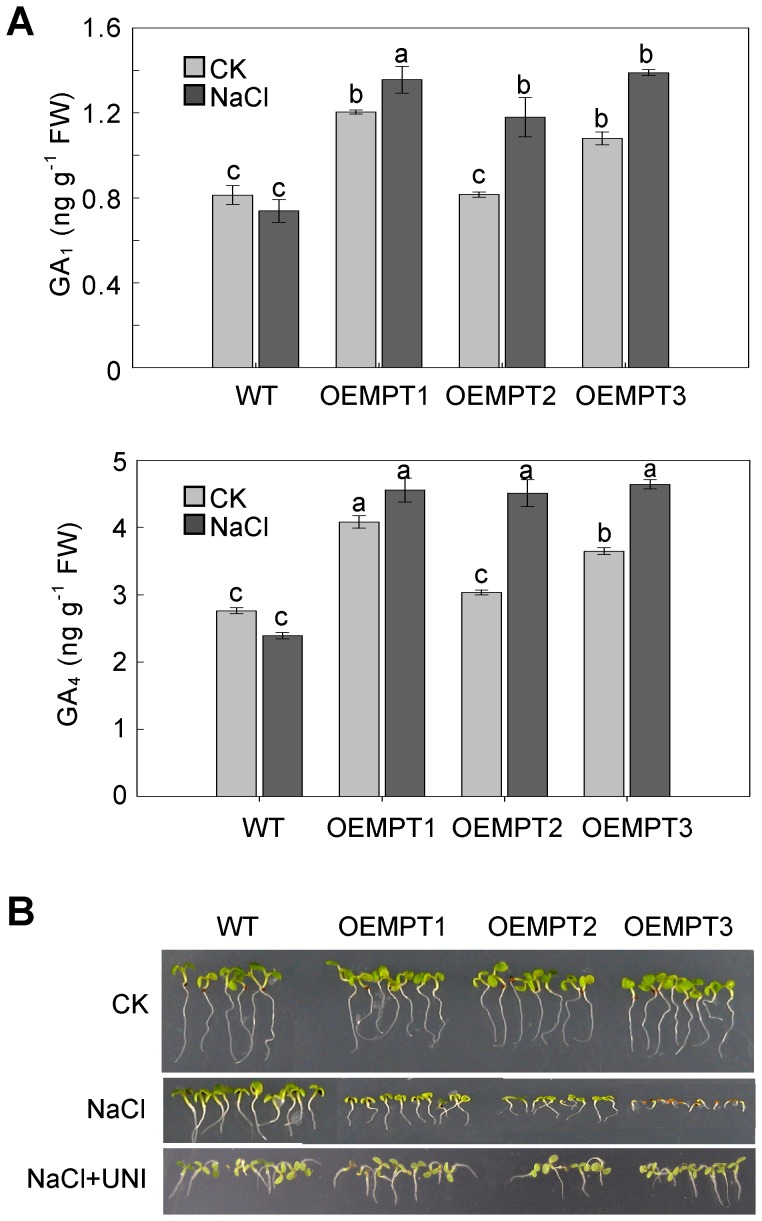
The role of gibberellin in AtMPT-modulated early responses to salt stress. (**A**) ELISA-detectable gibberellin (GA_1_ and GA_4_) content of 10-day-old wild type and overexpression lines grown under control and high salinity stress. Values represent the average of three replicates±SEM. Samples with different letters are significantly different: P<0.01 (a and c, b and c) or P<0.05 (a and b). (**B**) Germination and seedling establishment was observed on control and 150 mM NaCl supplemented with 10 nM uniconazole. The pictures were taken 10 days post-germination. OEMPTs, the *AtMPT* overexpressors.

To explore whether the bioactive gibberellin levels were affected by changes in the AtMPT expression, we determined by ELISA analysis the content of gibberellins, GA_1_ and GA_4_, which have been shown to play important roles in plant development in *A. thaliana* [38], [39]. As shown in Figure 6A, levels of ELISA-detectable gibberellins were elevated in the overexpressors after salt treatment. However, slightly lower ELISA-detectable gibberellin content was detected in wild-type plants after NaCl treatment. The significantly different trends between wild-type plants and the overexpression lines further support the notion that the metabolism of gibberellin is partially impaired in *AtMPT* overexpressors under salt stress.

To confirm this, we investigated the responsiveness of the overexpressors under salt stress to uniconazole, a gibberellin biosynthesis inhibitor [40], [41]. As shown in Figure 6B, the salt sensitivities of the overexpressors were restored to that of the wild-type after supplementing with 10 nM uniconazole during both the seed germination and seedling establishment stages. On the basis of these data, we suggest that gibberellins are involved in AtMPT-mediated early responses to salt stress in *A. thaliana.*

## Discussion

Plants growing in their native environments are constantly confronted by multiple types of stress. The cellular and molecular responses of plants to these stresses have been studied intensively [42], [43]. And we speculated that a relationship between abiotic stress tolerance and the energy status of plant cells might exist. As we know, cellular ATP is regenerated from ADP and phosphate in mitochondria, and the transport of phosphate from cytosol into the mitochondrial matrix is catalyzed by the mitochondrial phosphate transporter. However, because of the complexity and diversity of cell metabolism, the role of the mitochondrial phosphate transporter in plant response to abiotic stress is still poorly understood.

In our present study, we have characterized all three cDNAs encoding typical MPTs in *A. thaliana* (Figure 1 and S1). Their promoter-GUS and qRT-PCR analysis indicated different tissue-specific expression patterns (Figure 2). Remarkably, the expression level of *AtMPT3* was much higher compared with the other two *AtMPT* genes under both control and salt stress conditions (Figure 2 and 3). We also found that the *AtMPT3* overexpressors exhibited phenotypic abnormalities, such as late flowering, anomalous shape of rosette and cauline leaves, and reduced fertility, whereas the overexpressors of *AtMPT1* and *AtMPT2* grew normally as wild-type plants (Figure S6). Furthermore, among the three AtMPTs the amino acid sequence of AtMPT3 shows highest identity with MPTs from other species (Table S1). In fact, a similar conclusion has been based on complementation of a yeast mutant lacking the endogenous PiC, in which AtMPT3 was more effective than the other two MPT genes [17]. These data reveal that AtMPT3 might play a dominant role among three in development by influencing energy metabolism. A previous study reported that AtMPT2 and AtMPT3 have phosphate transport activity [17], but it remains to be seen whether AtMPT1 has the same function. Here, we show that the ATP content is higher in *AtMPT1* overexpressors than that in wild-type plants, and the phenotype of its overexpressors is similar to the overexpression lines of *AtMPT2* and *AtMPT3* under salt stress (Figure 3 and 4). Sequence analysis also reveals that AtMPT1 contains most of the critical residues (Figure S1). We thus speculate that AtMPT1 also has phosphate transport activity.

Several reports have pointed out that multiple mitochondrial phosphate transporters exist in yeast, humans and rice and play distinct roles [17], [44], [45]. For example in *Saccharomyces cerevisiae*, Pic2p is considered to be a minor form compared with MIR1. In addition, six rice MPTs display specific expression profiles, and *OsPT17* and *OsPT19* are regulated differently under hormone treatment conditions, suggesting various functions in rice plants. Based on the specific expression of *AtMPTs*, we can also propose that they function in different developmental stages or organs in *A. thaliana*.

Currently, modulation of cellular energy homeostasis is an attractive way for improving plant performance and yielding stability during environmental stress. In the mitochondrial CMSII mutant of *Nicotiana sylvestris*, increased pools of NAD and NADH are concomitant with increased resistance to ozone and tobacco mosaic virus [46], [47]. Elevated pools of NAD(P)H are also believed to account for the improved ROS stress-resistance of transgenic rice overexpressing an NADPH-dependent *Helminthosporium carbonum* (HC) toxin reductase-like gene [48]. We thus speculate a relationship between the energy status of plant cells and abiotic stress tolerance exists.

Here, we provide evidence that the *AtMPT* overexpressors impair the ability to adjust to salt stress because of the change of energy homeostasis during seed germination and seedling establishment stages (Figure 4), revealing that variable ATP levels might be linked to plant response to salt stress. Furthermore, the sensitivity of the overexpressors to salt stress could be recovered if the transport of excess energy from mitochondria into cytosol was blocked by atractyloside, supporting the idea that energy plays an important role in plant response to salt stress (Figure 4C).

In the present study, we also provide evidence that gibberellin homeostasis responding to salt stress is disturbed due to the overexpression of *AtMPTs*. Previously it was shown that the reduced gibberellin level causes high salinity stress tolerance by regulating DELLA proteolysis [29]. And transgenic *A. thaliana* plants overexpressing *DDF1*, a gene responsive to high salinity, improve responses to salt stress due to the repression of gibberellin synthesis, indicating that gibberellin plays an important role in salt stress tolerance [30]. For our study, we show that the expression of gibberellin metabolism genes and ELISA-detectable gibberellins are regulated by AtMPTs in response to salt stress (Figure 5 and 6A). For example, under salt stress conditions, the transcripts of *GA20ox and GA2ox* genes were significantly up- and down-regulated, respectively, presumably accounting for the elevated levels of ELISA-detectable gibberellins within the *AtMPT3* overexpressors. Previous studies have also pointed that gibberellin metabolism genes allow for differentially regulation during specific developmental stages and function cooperatively or individually to control gibberellin content during plant development [49]–[51]. Interestingly, the induction of *GA 2-oxidase 7* gene in *DDF1*-overexpressing *A. thaliana* reduces endogenous gibberellin levels, leading to growth repression for salt stress adaptation [30]. Thus, we speculate that in response to salt stress AtMPTs mainly affect the gibberellin deactivation enzymes, while the gibberellin synthesis genes might act coordinately to maintain gibberellin homeostasis of the *AtMPT3* overexpressors under control conditions. The results from applying uniconazole confirm that gibberellin affects AtMPT-mediated early responses to salt stress in *A. thaliana* (Figure 6B). All these observations can account for the fact that the overexpression of *AtMPT*s impacts gibberellin level, which in turn modulates the response to salt stress. Although the data resulted from three biological replicates, the ELISA assay used to detect the bioactive gibberellin levels might have given misleading results because of interfering compounds in the extracts. To substantiate the proposed relationship between gibberellins and AtMPTs, it will be necessary to analyze both bioactive and inactive gibberellin content using GCMS or LCMS approaches.

As we know, mitochondria are where a large amount of free energy is released and used for ATP production. In this process, the mitochondrial phosphate transporter takes charge of transporting phosphate from the cytosol into the mitochondrial matrix. To figure out the possible consequences resulting from the changes of ATP levels during salt stress, we surveyed the public transcriptome databases. As shown in Figure S7, these changed genes are putatively involved in a set of physiological, metabolic, and molecular events, including gibberellin metabolism. It was recently reported that an interaction between energy metabolism and gibberellin-mediated control of growth exists in *A. thaliana* [52]. Our results indicate that the gibberellin metabolism might be affected by the overexpression of AtMPTs via regulating ATP level (Figure 5). Furthermore, the high salinity sensitivity of the overexpressors was both recovered by exogenous application of atractyloside and uniconazole (Figure 4 and 6). Taken together, we propose that there is a possible link between energy availability impacted by AtMPTs and gibberellin metabolism in *A. thaliana* when response to salt stress.

## Materials and Methods

### Plant Materials, Growth Conditions, and Treatments

Plants of *Arabidopsis thaliana* (L.) Heynh. ecotype Columbia were germinated and grown on 1/2 MS medium supplemented with 1% (w/v) sucrose at 22°C in a growth chamber on an 18-hr light/6-hr dark cycle. For different stress treatments in RT-PCR, real time RT-PCR analysis and the measure of ATP and gibberellin content, uniformly developed 10-day-old seedlings were transferred onto filter paper soaked with water or 150 mM NaCl for 24 hours. For phenotype analysis, NaCl, mannitol, atractyloside (ATP/ADP carrier inhibitor) and uniconazole, were added to the 1/2 MS medium at different concentrations indicated in the text. In all experiments, at least 100 seeds were used per treatment. Seed germination and seedling establishment was tested on vertical 1.5% (w/v) agar plates. Each experiment was repeated three times.

### Generation of P*_MPTs_*::GUS Reporter Lines and MPTs Overexpression Lines

The pBI121 binary vector containing *35S::MPTs* and *P_MPTs_::GUS* were introduced into *Agrobacteriun tumefaciens* strain GV3101 and then transformed into *A. thaliana* by floral dipping [53]. The transgenic plants were screened on 1/2 MS medium containing 50 mg mL^−1^ kanamycin. The corresponding T_1_ transgenic seedlings segregated at a ratio of 3∶1 (resistant: sensitive) were selected to propagate T_2_ individuals, which were used for further analysis. PCR primers were designed with the Primer Premier 5 software and shown in Table S3.

### Computer Analysis

Hydrophobicity profiles were performed by TMpred (http://www.ch.embnet.org/software) and protein structures were showed by SWISS-MODEL (http://swissmodel.expasy.org). The public transcriptome databases were analyzed by the genome tool Genevestigator which is an *A. thaliana* microarray database and analysis toolbox. Biological processes in which the differentially expressed genes involved were analyzed by the gene ontology (GO) (http://omicslab.genetics.ac.cn/GOEAST/).

### Histochemical Localization of GUS

*P_MPTs_::GUS* transgenic lines were immersed in GUS staining solution (50 mM sodium phosphate buffer pH 7.0, 0.2% Triton X-100, 10 mM potassium ferrocyanide, 10 mM potassium ferricyanide, 1 mM X-gluc). Samples were vacuum infiltrated for 10 min and then incubated at 37°C overnight. The reaction was stopped by washing the samples with 70% ethanol.

### Quantitative Real-time Reverse Transcription-PCR Analysis

Total RNA was extracted from 10-old-day WILD-TYPE and transgenic plants grown on 1/2 MS-agar media using Trizol reagents (Invitrogen, Carlsbad, CA, USA) according to the manufacturer’s instruction. The RNA was subjected to DNase treatment using the DNA-free kit (TransGen), and cDNA was synthesized by PrimeScript reverse transcriptase with oligo dT primer using the PrimeScript RT Master Mix kit (Takara). All samples were brought to 10 uL in volume. SYBR Green real-time PCR master mix (Toyobo) and a Chromo 4 real-time PCR detector (Bio-Rad) were used. A range of five dilutions of the total cDNA was tested in the same conditions as the samples. The following standard thermal profile was used: 30 s at 95°C, 40 repeats of 5 s at 95°C, 10 s at 60°C and 15 s at 72°C, and a final stage of 55°C to 95°C to determine dissociation curves of the amplified products. Transcript levels of genes of interest (GI) within a cDNA were normalized to the respective transcript level of EF1-a. One dilution of the total cDNA from each sample was serially diluted to generate the standard curve. Analyses of the melting curves were performed to ensure amplification of one specific gene product. Real-time PCR was performed in triplicate, and data were presented as the means±SEM of at three independent experiments. The PCR primers were designed by the Beacon Designer 7.0 program to produce 75–200 bp products in length and shown in Table S4.

### Measurements of ATP, ADP, AMP Content and Calculation of Energy Charge

Adenosine phosphates were extracted from fresh 10-day-old *A. thaliana* seedlings with different treatments. Extractions and measurements were performed as described previously [9]. Data are means±SEM of triplicate experiments using different lines. The adenylate energy charge is calculated according to: Energy charge = ([ATP] +1/2 [ADP])/([ATP] + [ADP] + [AMP]). The energy charge modulates the activity of various metabolic sequences related to energy utilization and regeneration. When the energy charge is greater than 0.5, the activities of ATP-utilizing systems increased. At a lower energy charge than 0.5 in cells, ATP-regeneration systems are dominant.

### The Quantification of Endogenous Gibberellins by Enzyme Linked Immunosorbent Assay (ELISA)

Gibberellin content was measured by ELISA, as described by Shan *et al.* (2007) [54]. Sixteen seedlings were used per treatment (four independent lines and wild type). Briefly, samples were homogenized in liquid nitrogen and extracted in cold 80% methanol with butylated hydroxytoluene (1 mM) overnight at 4°C. Gibberellin extraction was performed in triplicate. After centrifugation at 10 000 g (4°C) for 20 min, the extracts were passed through a C_18_ Sep-Pak cartridge and dried in N_2_. The residues were dissolved in phosphate-buffered saline (PBS) (pH 7.4). An ovalbumin solution (10 mg ml^−1^) was added to each well to block the nonspecific binding after the 96-well microtitration plates had been coated with synthetic GA_1_-ovalbumin conjugates in NaHCO_3_ buffer (50 mmol l^−1^; pH 9.6) overnight at 37°C. Then horseradish peroxidase-labeled goat anti-rabbit immunoglobulins were added to each well and incubated for 1 h at 37°C. The enzyme reaction was carried out in the dark at 37°C for 15 min after the substrate (ortho phenylenediamine) had been added, and then was terminated using 3 mol l^−1^ H_2_SO_4_. Calculations using the ELISA data were performed for absorbances recorded at 490 nm. The cross-reactivity of antibodies raised against GA_1_-ovalbumin to GA_4_-ovalbumin was 32% under the analytical conditions used. The gibberellin analysis was performed three times with individual plant samples.

### Statistical Analysis

Data were subjected to Data Processing System (DPS) and significant differences between individual means established using a Student’s t test. Differences at the 5% level were considered significant and denoted by the lowercase letters among different groups.

## Supporting Information

Figure S1 **Amino acid sequence alignments of AtMPTs with the rice, yeast and bovine MPTs.**
(DOC)

Figure S2 **The high salinity stress tolerance of the other two independent** ***AtMPT*** **overexpressors (OEMPTs) lines.**
(DOC)

Figure S3 **Analysis of osmotic sensitivities of transgenic** ***A. thaliana*** **.**
(DOC)

Figure S4 **Impact of supplentmental calcium on the growth of the overexpressors.**
(DOC)

Figure S5 **Surveyed results using the genome tool Genevestigator.**
(DOC)

Figure S6 **Phenotypes of** ***AtMPT3*** **overexpressing plants.**
(DOC)

Figure S7 **Categories of biological processes for the AtMPT target genes.**
(DOC)

Table S1 **The identity (%) between AtMPT proteins and the rice, yeast and bovine MPTs.**
(DOC)

Table S2 **Names and annotations of target genes encoded the important signal transduction components.**
(DOC)

Table S3 **Primers used for plasmid construction in this study.**
(DOC)

Table S4 **Real-Time PCR primers used in this study.**
(DOC)
