# Supplementary material for: The Mitochondrial Phosphate Transporters Modulate Plant Responses to Salt Stress via Affecting ATP and Gibberellin Metabolism in Arabidopsis thaliana
Source: PLoS One. 2012 Aug 24;7(8):e43530. doi: 10.1371/journal.pone.0043530 (PMC3427375; doi:10.1371/journal.pone.0043530)
Supplement: Figure S1 — Amino acid sequence alignments of AtMPTs with the rice, yeast and bovine MPTs. (DOC) [file pone.0043530.s001.doc]

**Figure S1**

*Arabidopsis* MPT1

*Arabidopsis* MPT2

*Arabidopsis* MPT3

Rice MPT1

Rice MPT2

Rice MPT3

Rice MPT4

Rice MPT5

Rice MPT6

Yeast MPT

Bovine MPT-A

Bovine MPT-B

*Arabidopsis* MPT1

*Arabidopsis* MPT2

*Arabidopsis* MPT3

Rice MPT1

Rice MPT2

Rice MPT3

Rice MPT4

Rice MPT5

Rice MPT6

Yeast MPT

Bovine MPT-A

Bovine MPT-B

*Arabidopsis* MPT1

*Arabidopsis* MPT2

*Arabidopsis* MPT3

Rice MPT1

Rice MPT2

Rice MPT3

Rice MPT4

Rice MPT5

Rice MPT6

Yeast MPT

Bovine MPT-A

Bovine MPT-B

*Arabidopsis* MPT1

*Arabidopsis* MPT2

*Arabidopsis* MPT3

Rice MPT1

Rice MPT2

Rice MPT3

Rice MPT4

Rice MPT5

Rice MPT6

Yeast MPT

Bovine MPT-A

Bovine MPT-B

*

*

*

*

*

*

*

*

*

*

*

*

*

*

*

*

*

*

*

*

*

*

*

*

*

*

*

*

*

*

*

*

*

*

*

*

*

*

*

*

*

*

*

*

*

*

*

*

*

*

*

I

II

III

IV

V

VI

42

91

102

93

93

94

4

96

60

41

90

89

145

198

209

200

200

201

107

203

163

149

197

196

247

300

311

302

302

303

209

305

265

255

299

298

309

363

375

368

369

360

278

374

323

312

363

362

MTRVKSKLDEE---------------------LSSPWFYTVCTMGGMLSAGTTHLAITPLDVL

MSDSSRSLIPSFLYSSDHRL--------FQATTMSTHLKSQPLISPTNSSVSSNGTSFAIATPNE---KVEMYSPAYFAACTVAGMLSCGITHTAITPLDVI MESPKNSLIPSFLYSSSSSPRSFLLDQVLNSNSNAAFEKSPSPAPRSSPTSMISRKNFLIASPTEPGKGIEMYSPAFYAACTFGGILSCGLTHMTVTPLDLV MALSDRSRESLLPSFLYTSSAAR---------SFATGATRFPSPVSPASPAPGAVGGGAPISIQAPRE---KIEMYSPAFYAACTAGGIASCGLTHMAVTPLDLV MALSDRSRESLLPSFLYSTAGAR---------PYTAGGGGAAFRL-PAAAAPGGVGGGAPIEIQAPRE---KIEMYSPAFYAACTAGGIASCGLTHMAVTPLDLV MAVVSESSRKALLPSFLYAAPAAS------------SSPSFAAAAAVGVRGVPAPSLAGPAVWARSPREPAGKIEMYSPAFYAACTAGGIASCGLTHMTVTPLDLV

MLVS-------------

MASPDKVGCSPEPLPLDRLLAELAAN-----------AERLGRRWEAALRKRGREGARAAGVGLEEGRKADS-AAMQLHTPLFYATCALGGLLSTGLTHLAVTPLDLV

MPEMGARGGEAGAARVANGGGGGGTG---GMRLFSPEYYALCTGGGMLAAGATHLAITPLDVL

MSVSAAPAIP-------------------QYSVSDYMKFALAGAIGCGSTHSSMVPIDVV

MFSSVAHLARANPFNTPHLQ------------LLHDGLGDLRSNPPGPTGQPRRPRNLAAAAVEEQ-YSCDYGSGRFFILCGLGGIISCGTTHTALVPLDLV

MFSSVAHLARANPFNTPHLQ------------LLHDGLGDLRSNPPGPTGQPRRPRNLAAAAVEE-—YSCEFGSAKYYALCGFGGVLSCGLTHTAVVPLDLV

KVNMQVNPVKYNS-IPSGFSTLLREHGHSYLWRGWSGKLLGYGVQGGCRFGLYEYFKTLYSDVL----PNHNRTSIYFLSSASAQIFADMALCPFEAIKVRVQTQPMF KCNMQIDPLKYKN-ITSAFKTTIKEQGLKGFTRGWSPTLLGYSAQGAFKYGLYEYAKKYYSDIVGPEYAAKYKTLIYLAGSASAEIVADVALCPMEAVKVRVQTQPGF KCNMQIDPAKYKS-ISSGFGILLKEQGVKGFFRGWVPTLLGYSAQGACKFGFYEYFKKTYSDLAGPEYTAKYKTLIYLAGSASAEIIADIALCPFEAVKVRVQTQPGF KCNMQIDPAKYKS-ITSGFGVLLKEQGPRGFFRGWVPTLLGYSAQGACKFGFYEFFKKYYSDIAGPEYAQKYKTLIYLAGSASAEVIADVALCPFEAVKVRVQTQPGF KCNMQIDPAKYKS-ITSGFGILAKEQGVRGFFRGWVPTLLGYSAQGACKFGFYEFFKKYYSDIAGPEYAQKYKTLIYLAGSASAEVIADVALCPFEAVKVRVQTQPGF KCNMQIDPAKYKS-ISSGFGVLLKEQGARGFFRGWVPTLLGYSAQGACKFGFYEFFKKYYSDIAGPEYAAKYKTLIYLAGSASAEVIADIALCPMEAVKVRVQTQPGF ----QIDPTKYKS-TTSAFGVVMREQGARGFYRGWAPTFLGYSAQGAFKYGLYEVFKKEYADMAGPEYAARYKTLIYLAGSATAEVAADVALCPMEAVKVRVQTQPGY KCNMQVDPGKYRD-ISSGFGVLLQEQGLGGFFKGWMATLVGYSSQGACKFGFYEFFKKCYSDIAGPEHAEKWKTFIYLAASASAEMIADVALCPMEAVKVRVQTQPGF KVNMQVNPMKYNS-IFSGLNILVKEEGASSLWRGWAGKFFGYGFQGGCKFGLYEYFKKKYSDVL----VDRNKSTIYFISSASAQIIADVALCPFESVKVRVQTQPMF KTRIQLEPTVYNKGMVGSFKQIIAGEGAGALLTGFGPTLLGYSIQGAFKFGGYEVFKKFFIDNLGYDTASRYKNSVYMGSAAMAEFLADIALCPLEATRIRLVSQPQF

KCRMQVDPQKYKG-IFNGFSVTLKEDGVRGLAKGWAPTFIGYSMQGLCKFGFYEVFKVLYSNMLGEENTYLWRTSLYLAASASAEFFADIALAPMEAAKVRIQTQPGY

KCRMQVDPQKYKG-IFNGFSVTLKEDGVRGLAKGWAPTFIGYSMQGLCKFGFYEVFKVLYSNMLGEENTYLWRTSLYLAASASAEFFADIALAPMEAAKVRIQTQPGY

AKGLLDGFPRVYRSEGLAGFHRGLFPLWCRNLPFSMVMFSTFEQSVEFIYQKIIQKRKQDCSKAQQLGVTCLAGYTAGAVGTIISNPADVVLSSLYNNKAKN------

ARGLSDGLPKIIKSEGFRGLHKGLVPLWGRQIPYTMMKFATFENTVELIYKKVMPTPKEECSKPVQLGVSFAGGYIAGIFCAIISHPADNLVSFLNNSKGAT------

ARGMSDGFPKFIKSEGYGGLYKGLAPLWGRQIPYTMMKFASFETIVEMIYKYAIPNPKSECSKGLQLGVSFAGGYVAGVFCAIVSHPADNLVSFLNNAKGAT------

ARGLSDGLPKFVRSEGALGLYKGIVPLWGRQIPYTMMKFASFETIVEMIYKHAVPVPKSECSKSFQLGISFAGGYIAGVFCAIVSHPADNLVSFLNNAKGAT------

ARGLGDGLPKFIKSEGALGLYKGIVPLWGRQIPYTMMKFASFETIVEQIYKHAVPVPKSECSKSFQLGISFAGGYIAGVFCAIVSHPADNLVSFLNNAKGAT------

ARGLSDGLPKFVKAEGYAGLYKGIVPLWGRQIPYTMMKFASFETVVEMIYKYAIPAPKSECSKPLQLGVSFAGGYIAGVFCAIVSHPADNLVSFLNNAKGAT------

ARGLSDGFPKIVRNESYAGLFRGLVPLWGRQIPYTMMKFATYENIVEMAYKHLIPTPKEQCSKPLQLGVSFGSGYIAGVFCAAVSHPADNLVSFLNNSKGAT------

ARCLTDGFPKIVQSEGAFGLYKGLLPLWGRQVPYTMVKFACFETIVELVYKHAVPKPKDECSKPLQLAVSFAGGYIAGVFCAAISHPADNLVSFLNNAKGGT------

AKGLIDGFPRVYATEGLSGFYKGLLPLWGRNLPFSMLMFSTFEHTVDILYRNVIQKKKEDCSTMQQLGATCLAGYISGAVGTVVSNPADNIVSSLYNKKAKN------

ANGLVGGFSRILKEEGIGSFYSGFTPILFKQIPYNIAKFLVFERASEFYYGFAGP--KEKLSSTSTTLLNLLSGLTAGLAAAIVSQPADTLLSKVNKTKKAPGQSTVG

ANTLRDAAPKMYKEEGLKAFYKGVAPLWMRQIPYTMMKFACFERTVEALYKFVVPKPRSECSKPEQLVVTFIAGYIAGVFCAIVSHPADSVVSVLNKEKGSS------

ANTLRDAAPKMYKEEGLKAFYKGVAPLWMRQIPYTMMKFACFERTVEALYKFVVPKPRSECSKPEQLVVTFIAGYIAGVFCAIVSHPADSVVSVLNKEKGSS------

-VLQAVRNIGFVGLFTRSLPVRITIVGPVITLQWFFYDAIKVLSGFPTSGGVKKPVDAAKLSV--------

-VADAVKRLGLWGMLTRGLPLRIFMIGTLTGAQWVIYDAVKVLAGLPTTGGASPATALAP------SVSA-

-VGDAVKKIGMVGLFTRGLPLRIVMIGTLTGAQWGLYDAFKVFVGLPTTGGVAPAPAIAAT---EAKA---

-VGDAVKKLGLWGLFTRGLPLRIVMIGTLTGAQWGIYDAFKVMVGLPTTGGVTPAPGAAEK---ALQASA-

-VGDAVKKLGLWGLFTRGLPLRIVMIGTLTGAQWGIYDAFKVMVGLPTTGGVTPAPSTSDA---GLKAVSA

-VGDAVNKLGMWGLFTRGLPLRIVMIGTLTGAQWGLYDAFKVMVGLPTTGGVAPTPAK-------------

-VGDAVKNLGLWGLFTRGLPLRILMIGTLTGTQWVIYDSFKVMVGLPTTGGAPAPAAIPIGELAELKASA-

-MADAVRTLGVWGLLTRGLPLRIIMVGTLTGAQWATYDAFKVFVGLPTSGGFIPSPAATDLRQVDHEKRS-

-IIDAVKSIGFRGLFTRSLPVRITLVGPVITMQWFFYDTIKILTGLPTSGGLPRELEEV------------

LLAQLAKQLGFFGSFA-GLPTRLVMVGTLTSLQFGIYGSLKSTLGCPPTIEIGGGGH--------------

-ASQVLKRLGFKGVWK-GLFARIIMIGTLTALQWFIYDSVKVYFRLPRP----PPPEMPESLKKKLGLTQ-

-ASQVLKRLGFKGVWK-GLFARIIMIGTLTALQWFIYDSVKVYFRLPRP----PPPEMPESLKKKLGLTQ-

**Figure S1. Amino acid sequence alignments of AtMPTs with the rice, yeast and bovine MPTs.** Asterisks represent amino acids conserved in all proteins, and gray shadings indicate amino acid residues conserved among three *Arabidopsis thaliana* MPTs. Dashes denote gaps introduced to facilitate the alignment. Orange shadings and roman numerals denote the potential transmembrane domains. Arrowheads indicate the important residues for the phosphate transport function of MPTs as identified by experiments in bovine or yeast.
