# Supplementary material for: The Mitochondrial Phosphate Transporters Modulate Plant Responses to Salt Stress via Affecting ATP and Gibberellin Metabolism in Arabidopsis thaliana
Source: PLoS One. 2012 Aug 24;7(8):e43530. doi: 10.1371/journal.pone.0043530 (PMC3427375; doi:10.1371/journal.pone.0043530)
Supplement: Figure S2 — The high salinity stress tolerance of the other two independent AtMPT overexpressors (OEMPTs) lines. (DOC) [file pone.0043530.s002.doc]

**Figure S2**

**A**

**B**


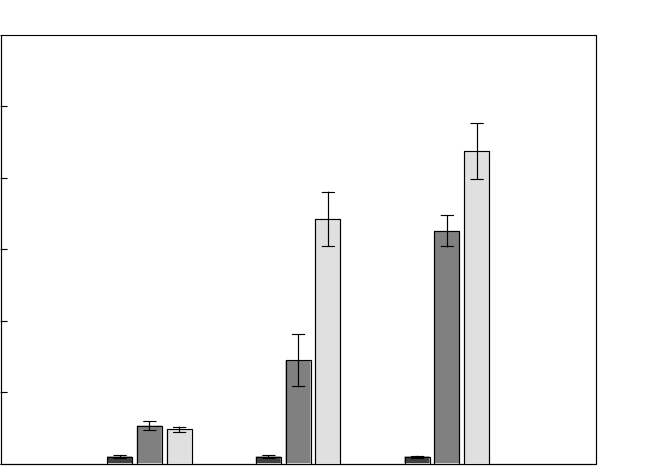


0

10

20

30

40

50

60

L3

L4

L9

L12

L15

L17

WT

WT

WT

Relative transcript level

WT L3 L4 L9 L12 L15 L17


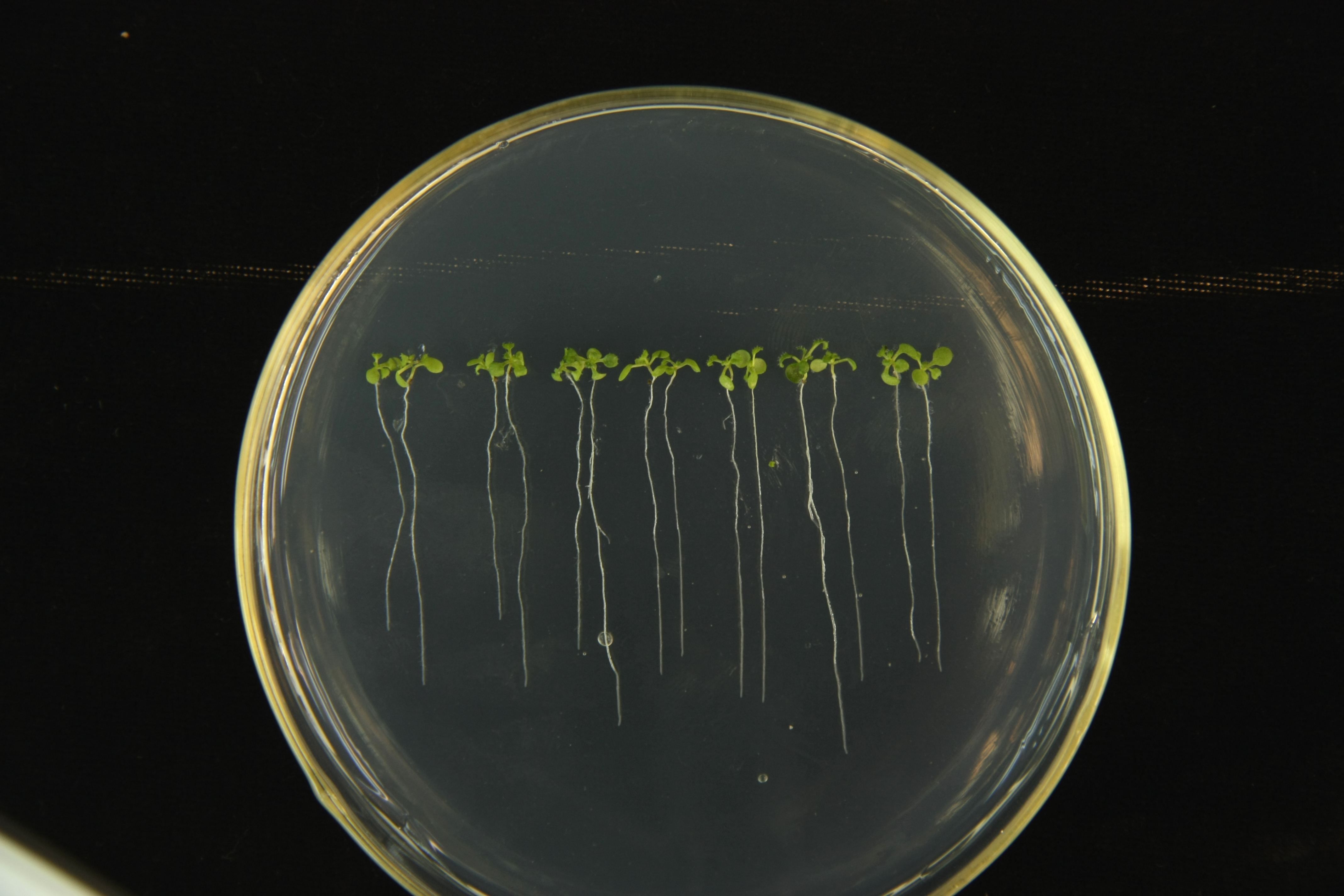


150 mM NaCl

CK

OEMPT1

OEMPT2

OEMPT3


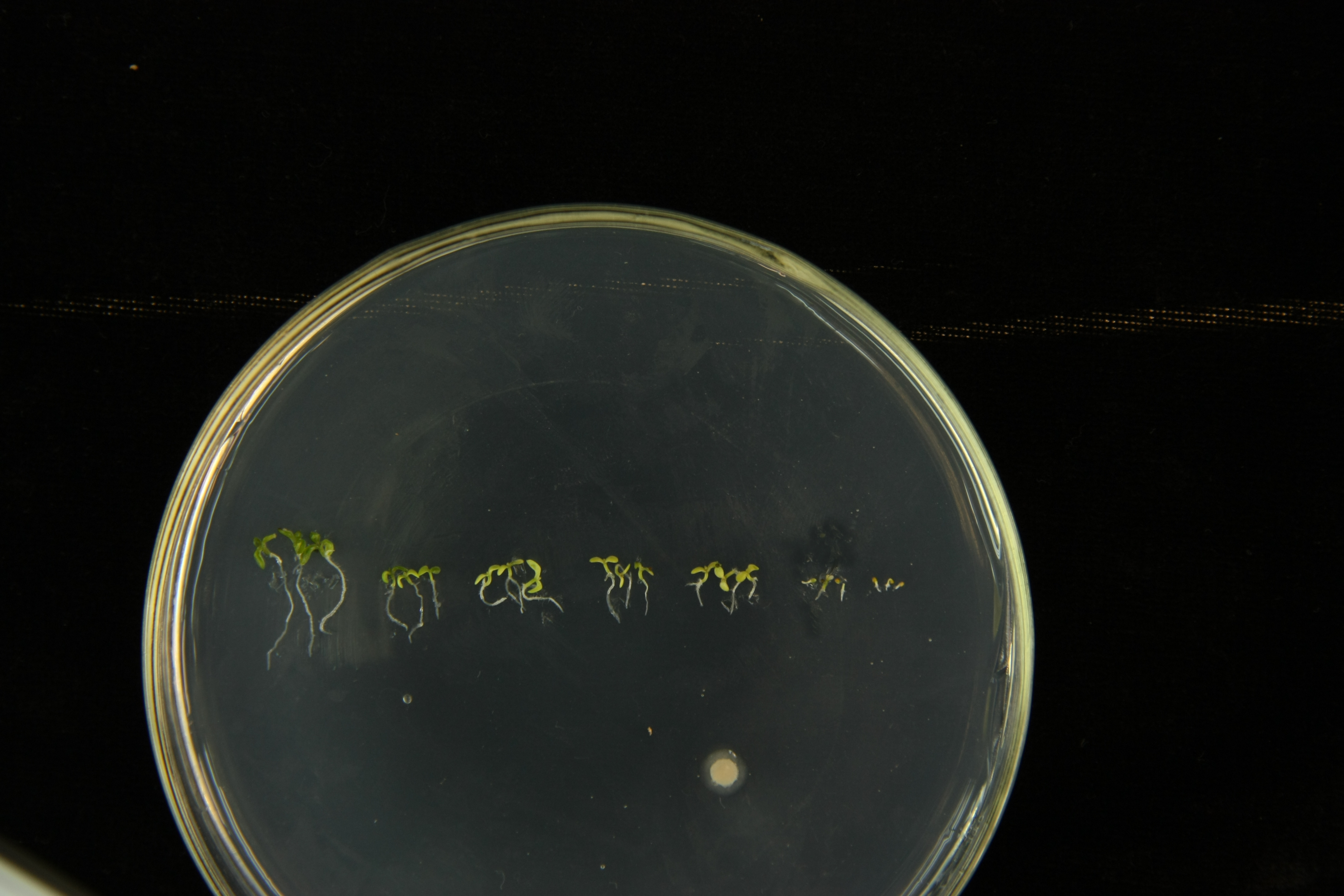


**Figure S2.** **The high salinity stress tolerance of the other two independent *AtMPT* overexpressors (OEMPTs) lines.** (**A**) RT-PCR analyzed *AtMPT* transcripts in wild-type plants and overexpressing plants (L1-3, L1-4, L2-9, L2-12, L3-15 and L3-17). The transcript level in the wild-type sample for transgenic plants overexpressing *AtMPTs* under control condition was set to 1, respectively. And the other levels were calculated relative to the corresponding value. EF1-α was used as a reference for template concentration. Values are means ±SEM of three replicates. (**B**) Wild type and overexpressing seedlings (L1-3, L1-4, L2-9, L2-12, L3-15 and L3-17) germinated and grown under control and salt stress. The pictures were taken after germination for 10 days. OEMPTs, the *AtMPT* overexpressors.
