# Supplementary material for: The Mitochondrial Phosphate Transporters Modulate Plant Responses to Salt Stress via Affecting ATP and Gibberellin Metabolism in Arabidopsis thaliana
Source: PLoS One. 2012 Aug 24;7(8):e43530. doi: 10.1371/journal.pone.0043530 (PMC3427375; doi:10.1371/journal.pone.0043530)
Supplement: Figure S3 — Analysis of osmotic sensitivities of transgenic A. thaliana . (DOC) [file pone.0043530.s003.doc]

**Figure S3**

CK

285 mM mannitol

WT OEMPT1 OEMPT2 OEMPT3


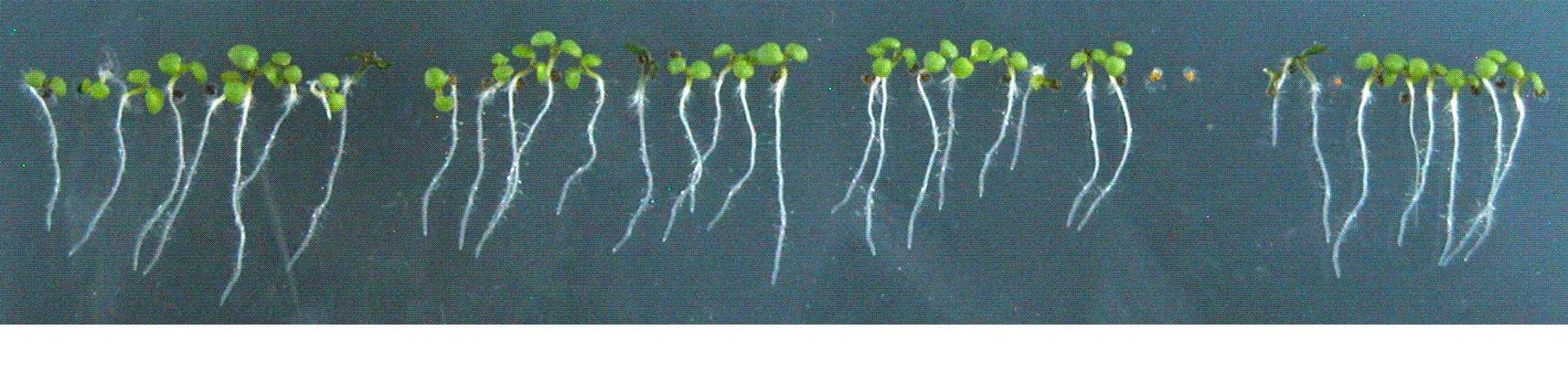

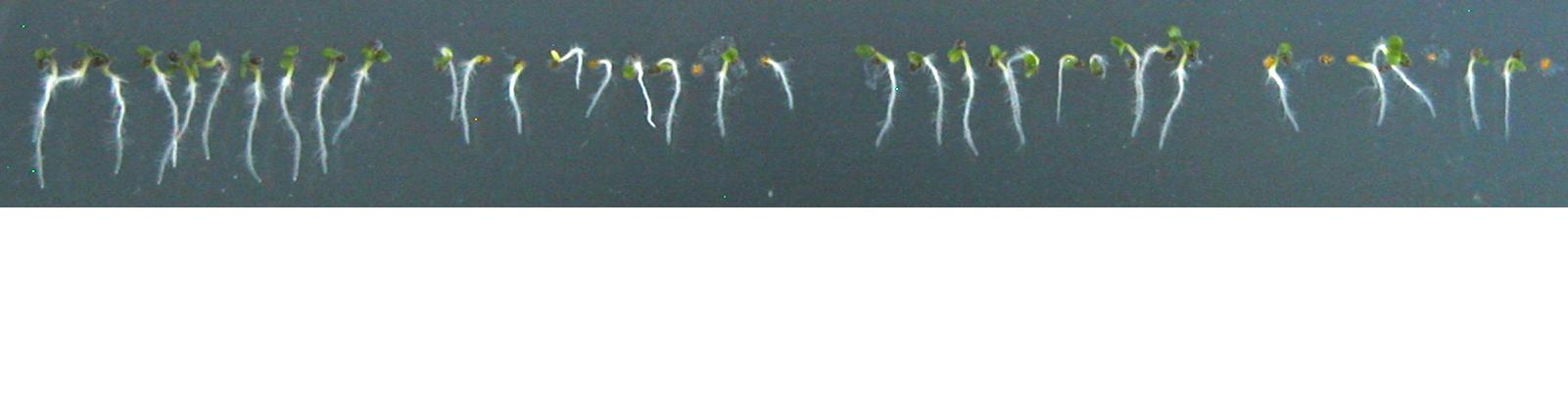


**Figure S3. Analysis of osmotic sensitivities of transgenic *A. thaliana*.** Analysis of osmotic sensitivities of transgenic *A. thaliana* overexpressing *AtMPTs* treated with 285 mM mannitol which led to the same osmotic potential with 150 mM NaCl. Plants photographed after germination for 10 days. OEMPTs, the *AtMPT* overexpressors.
