# Supplementary material for: The Mitochondrial Phosphate Transporters Modulate Plant Responses to Salt Stress via Affecting ATP and Gibberellin Metabolism in Arabidopsis thaliana
Source: PLoS One. 2012 Aug 24;7(8):e43530. doi: 10.1371/journal.pone.0043530 (PMC3427375; doi:10.1371/journal.pone.0043530)
Supplement: Figure S4 — Impact of supplentmental calcium on the growth of the overexpressors. (DOC) [file pone.0043530.s004.doc]

**Figure S4**


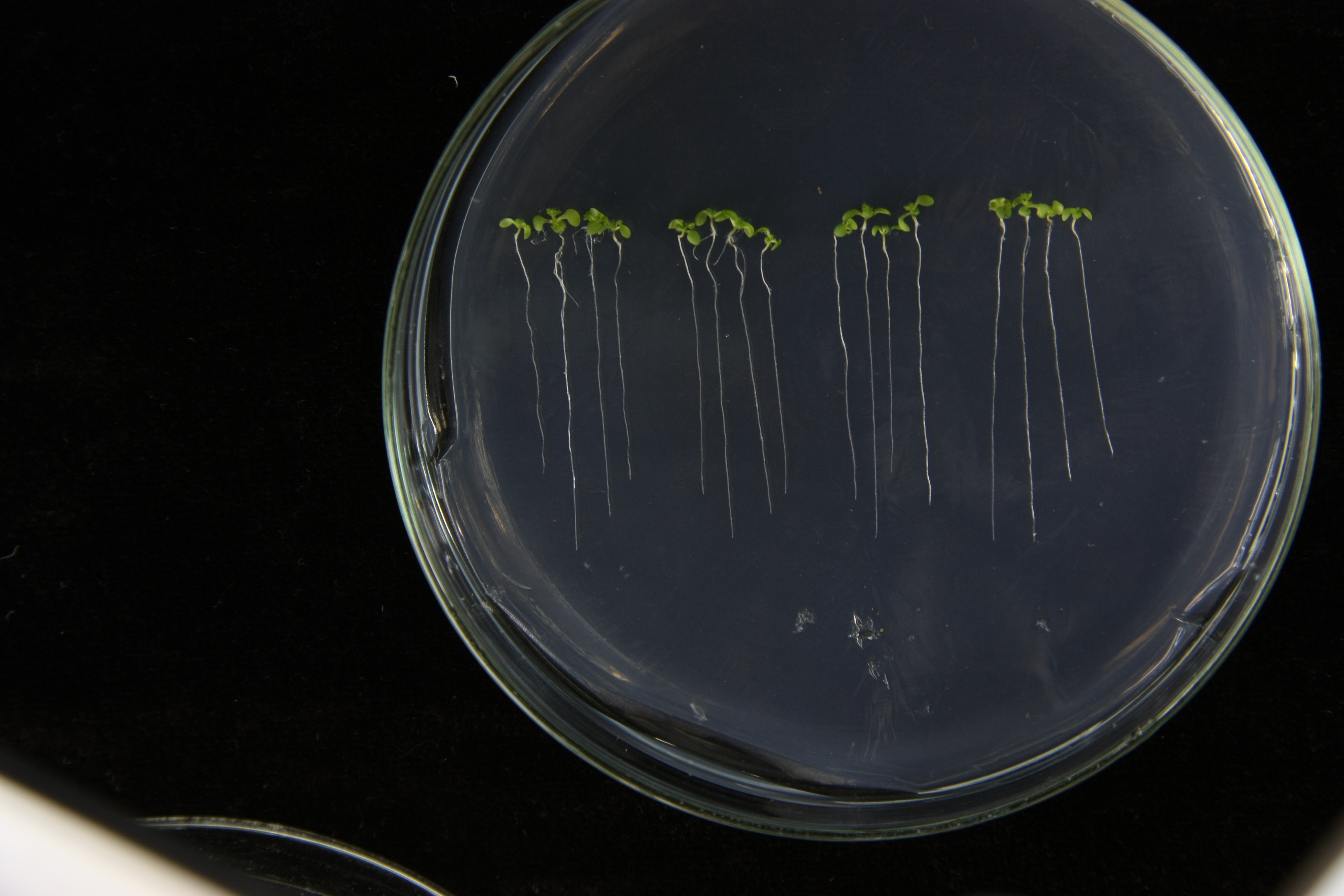

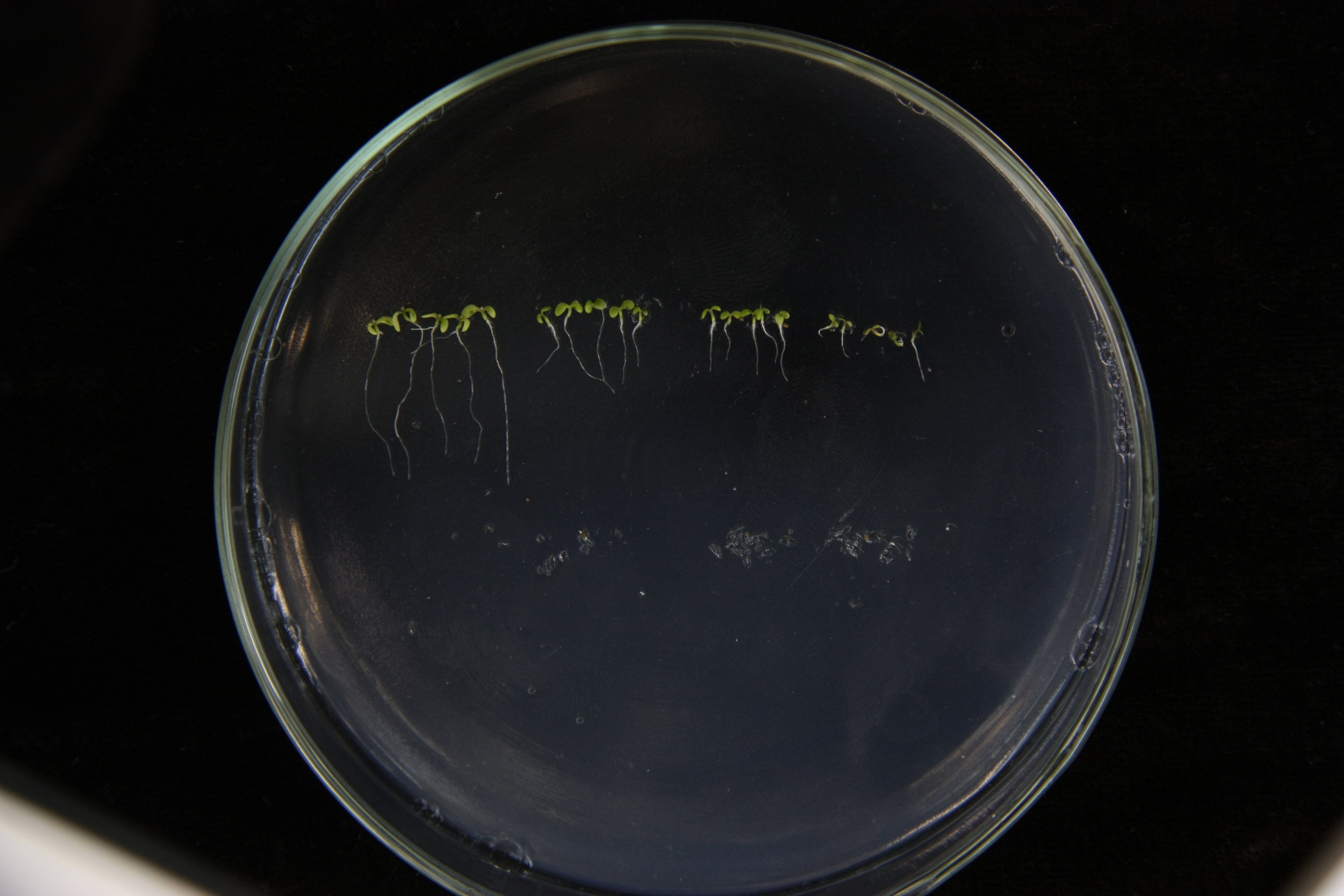


WT

OEMPT1

OEMPT2

OEMPT3


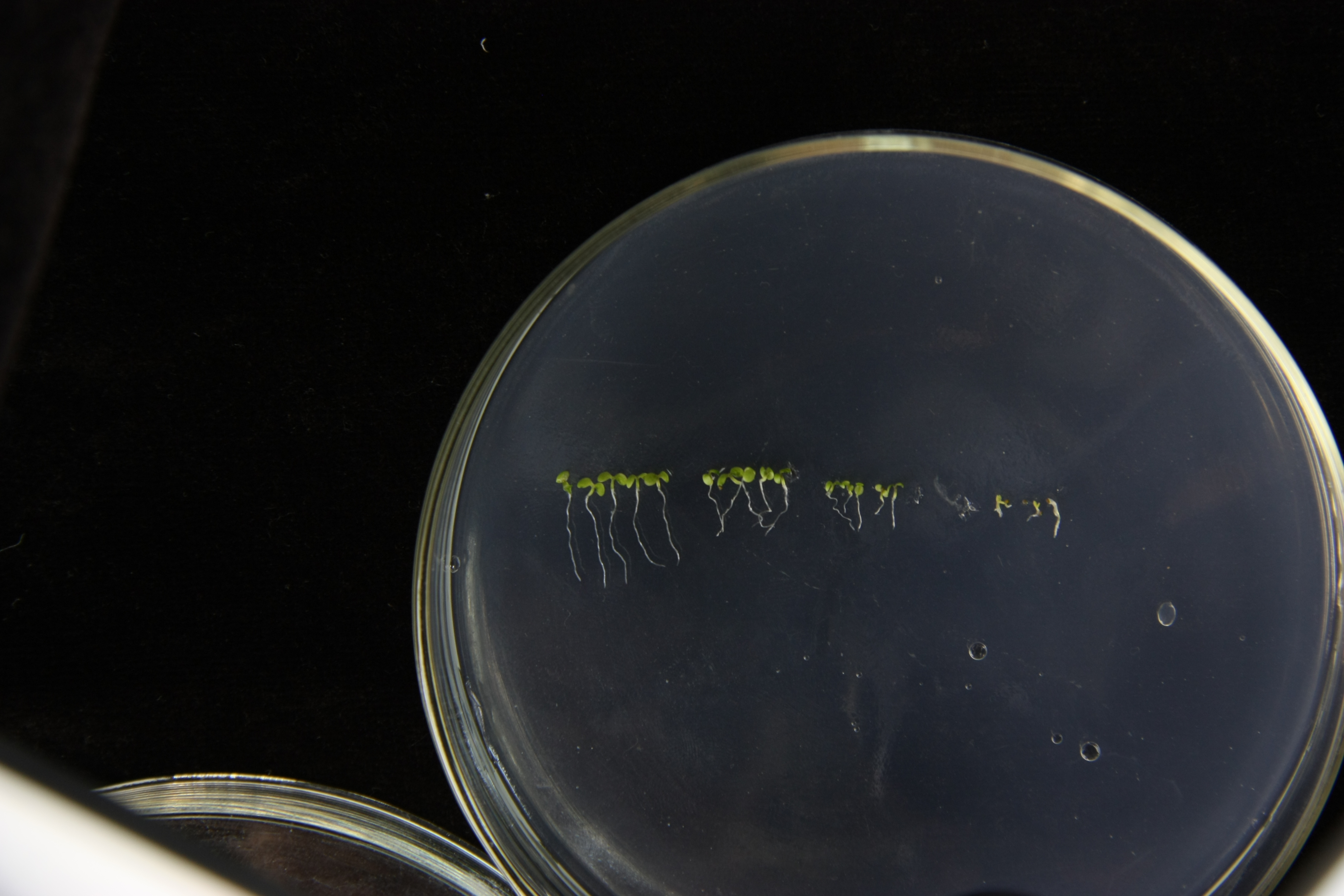

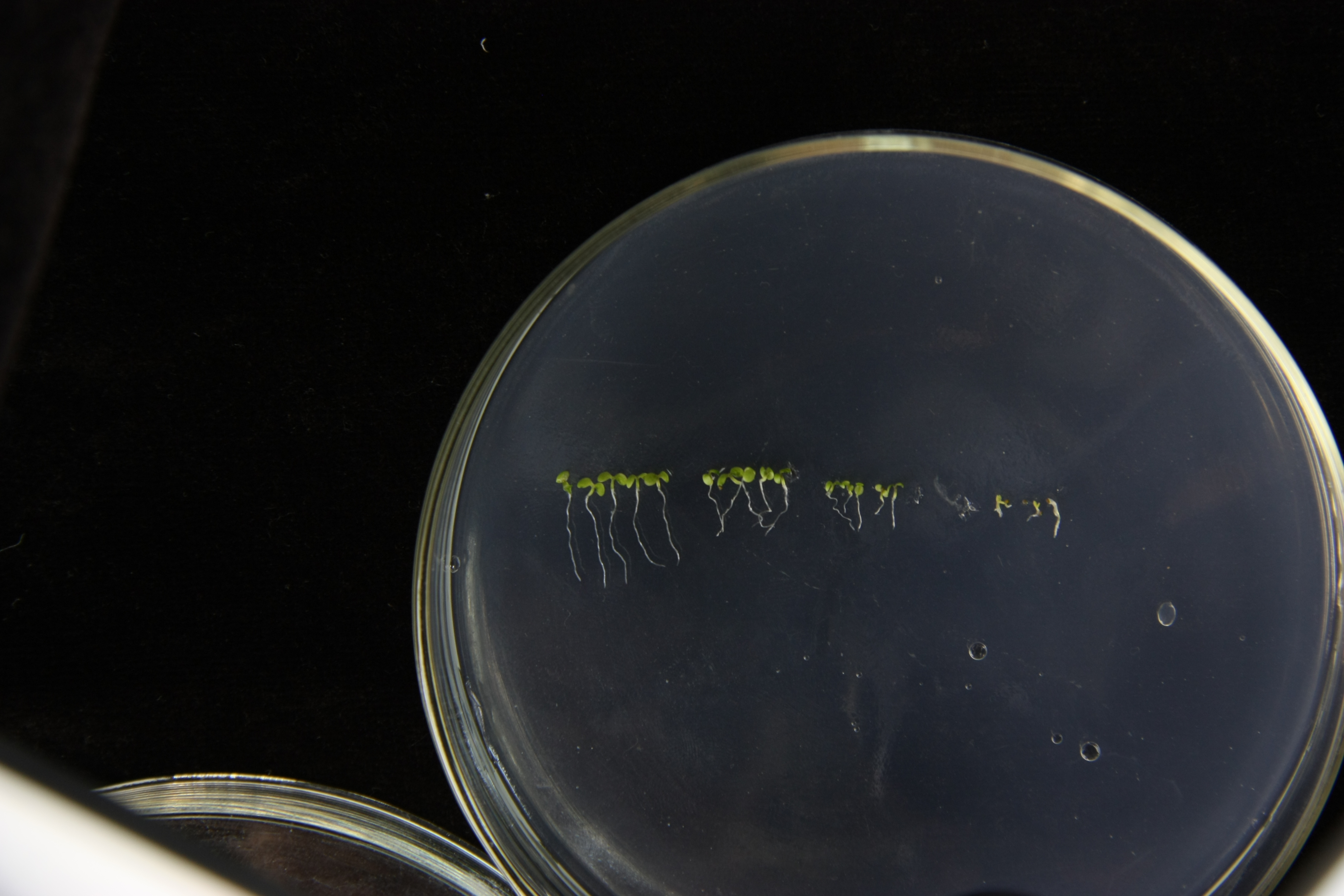

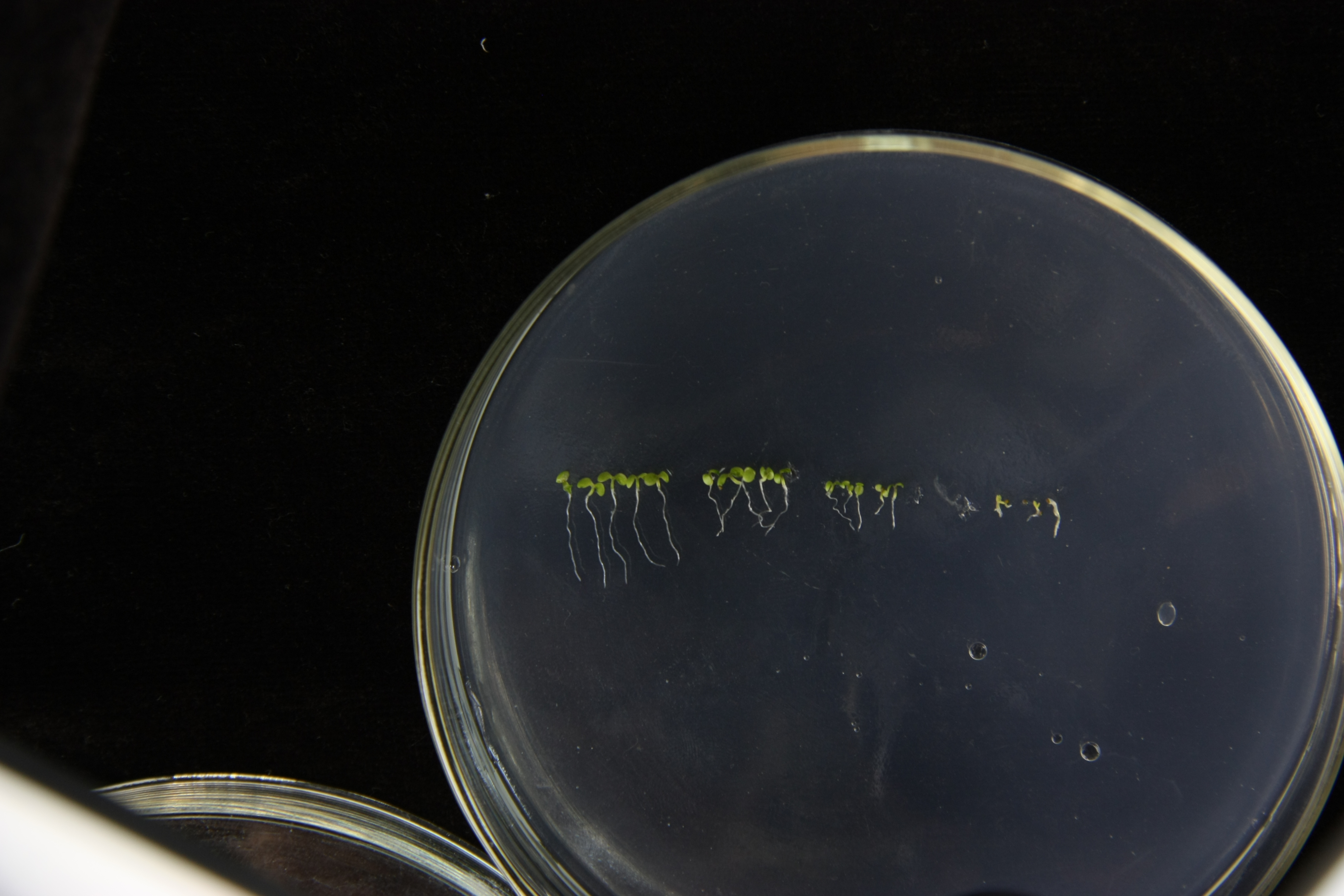

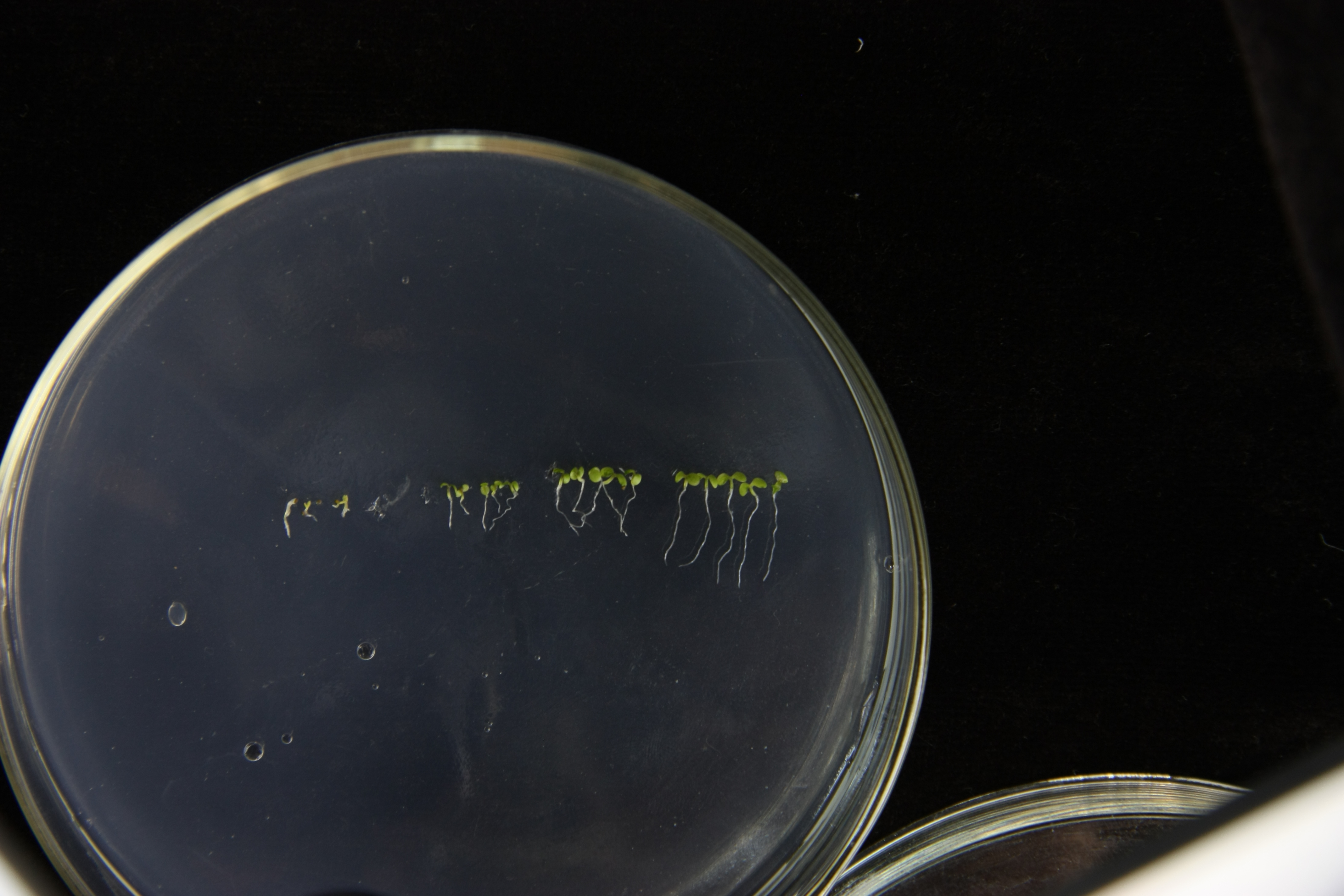

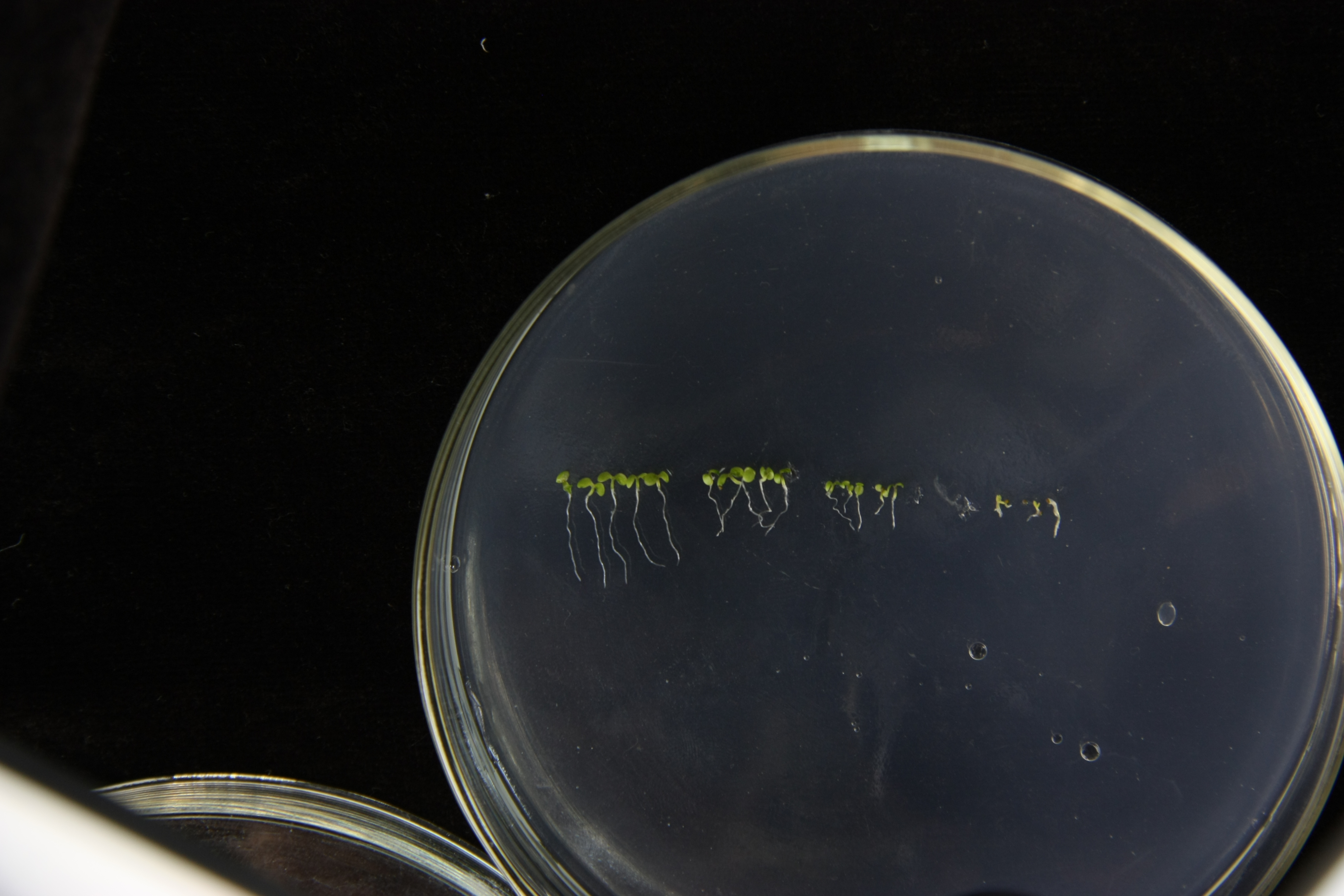

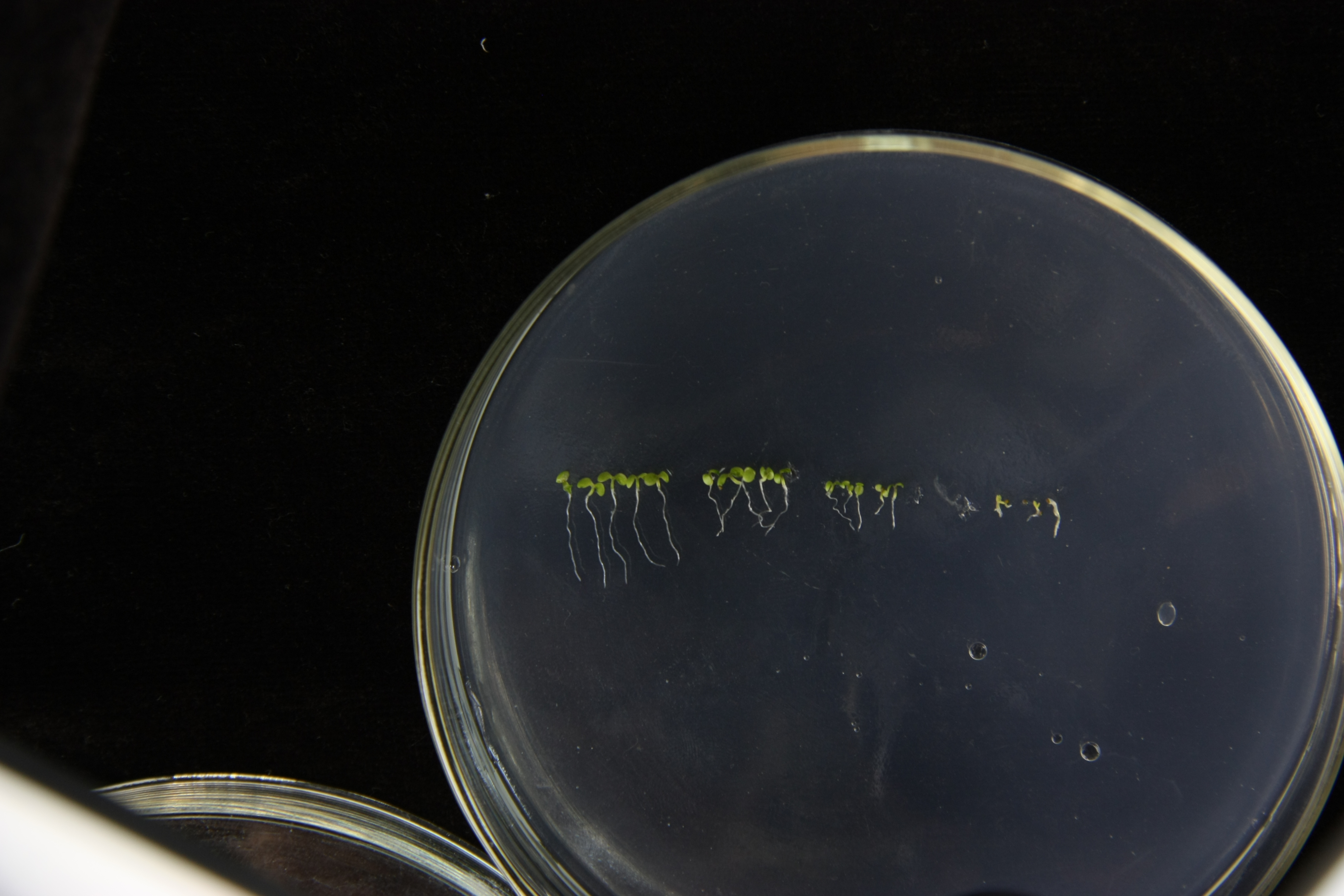

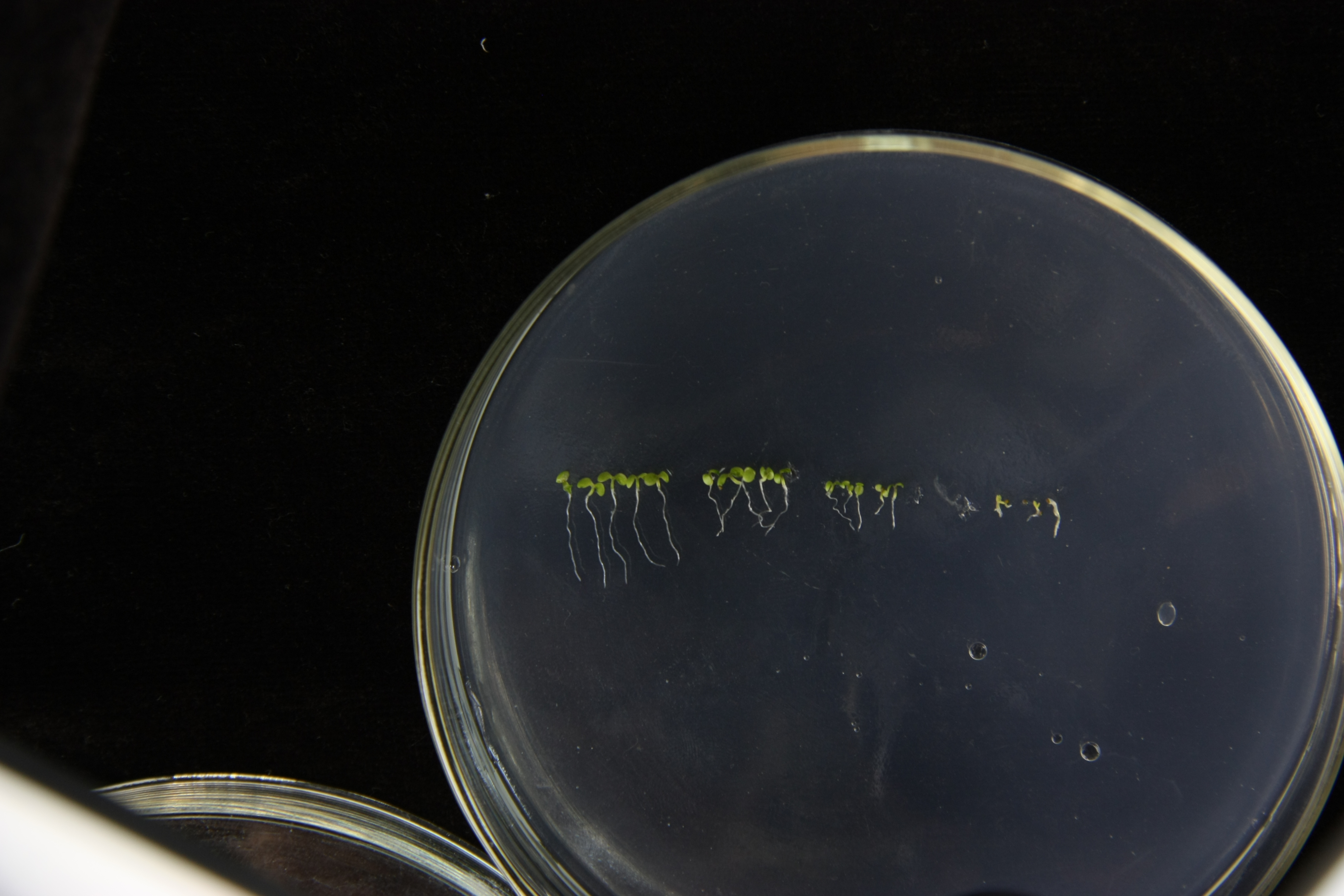


150 mM NaCl

150 mM NaCl + 7.5 mM CaCl_2_

CK

**Figure S4. Impact of supplentmental calcium on the growth of overexpressors.** Wide type and overexpressors germinated on 1/2 MS medium or 1/2 MS medium containing 150mM NaCl and 150 mM NaCl plus 7.5 mM CaCl_2_, respectively. Plants photographed after germination for 10 days. OEMPTs, the *AtMPT* overexpressors.
