# Supplementary material for: The Mitochondrial Phosphate Transporters Modulate Plant Responses to Salt Stress via Affecting ATP and Gibberellin Metabolism in Arabidopsis thaliana
Source: PLoS One. 2012 Aug 24;7(8):e43530. doi: 10.1371/journal.pone.0043530 (PMC3427375; doi:10.1371/journal.pone.0043530)
Supplement: Figure S5 — Surveyed results using the genome tool Genevestigator. (DOC) [file pone.0043530.s005.doc]

**Figure S5**


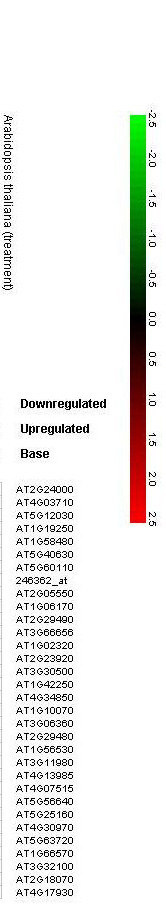

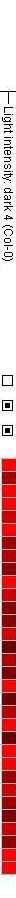

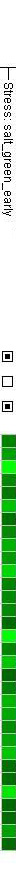

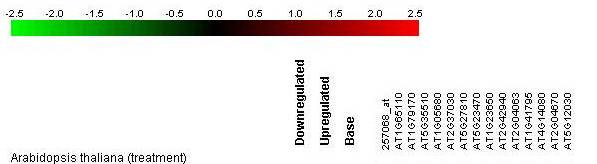

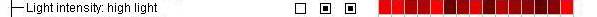

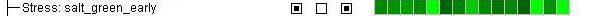


**A**


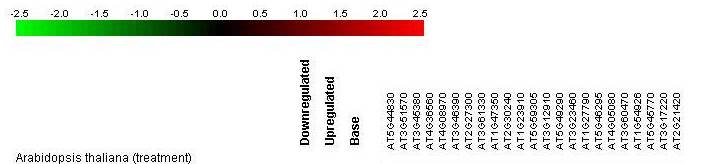

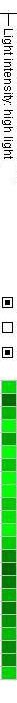

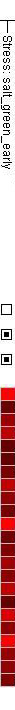

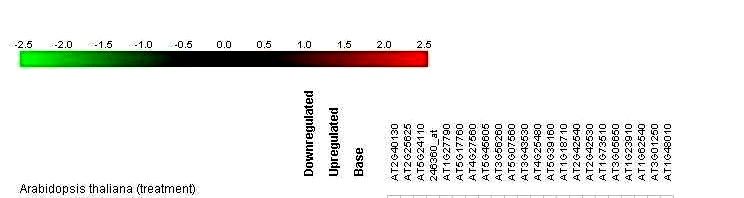

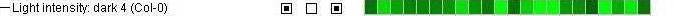

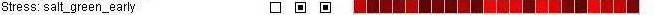


**B**

**Figure S5. Surveyed results using the genome tool Genevestigator.** (**A**) 47 salt-reduced genes that are activated by dark (cause energy decrease) and light (cause energy accumulation). (**B**) 45 salt-induced genes that are repressed by dark (cause energy decrease) and light (cause energy accumulation).
