# Supplementary material for: The Mitochondrial Phosphate Transporters Modulate Plant Responses to Salt Stress via Affecting ATP and Gibberellin Metabolism in Arabidopsis thaliana
Source: PLoS One. 2012 Aug 24;7(8):e43530. doi: 10.1371/journal.pone.0043530 (PMC3427375; doi:10.1371/journal.pone.0043530)
Supplement: Figure S6 — Phenotypes of AtMPT3 overexpressing plants. (DOC) [file pone.0043530.s006.doc]

**Figure S6**

**F**


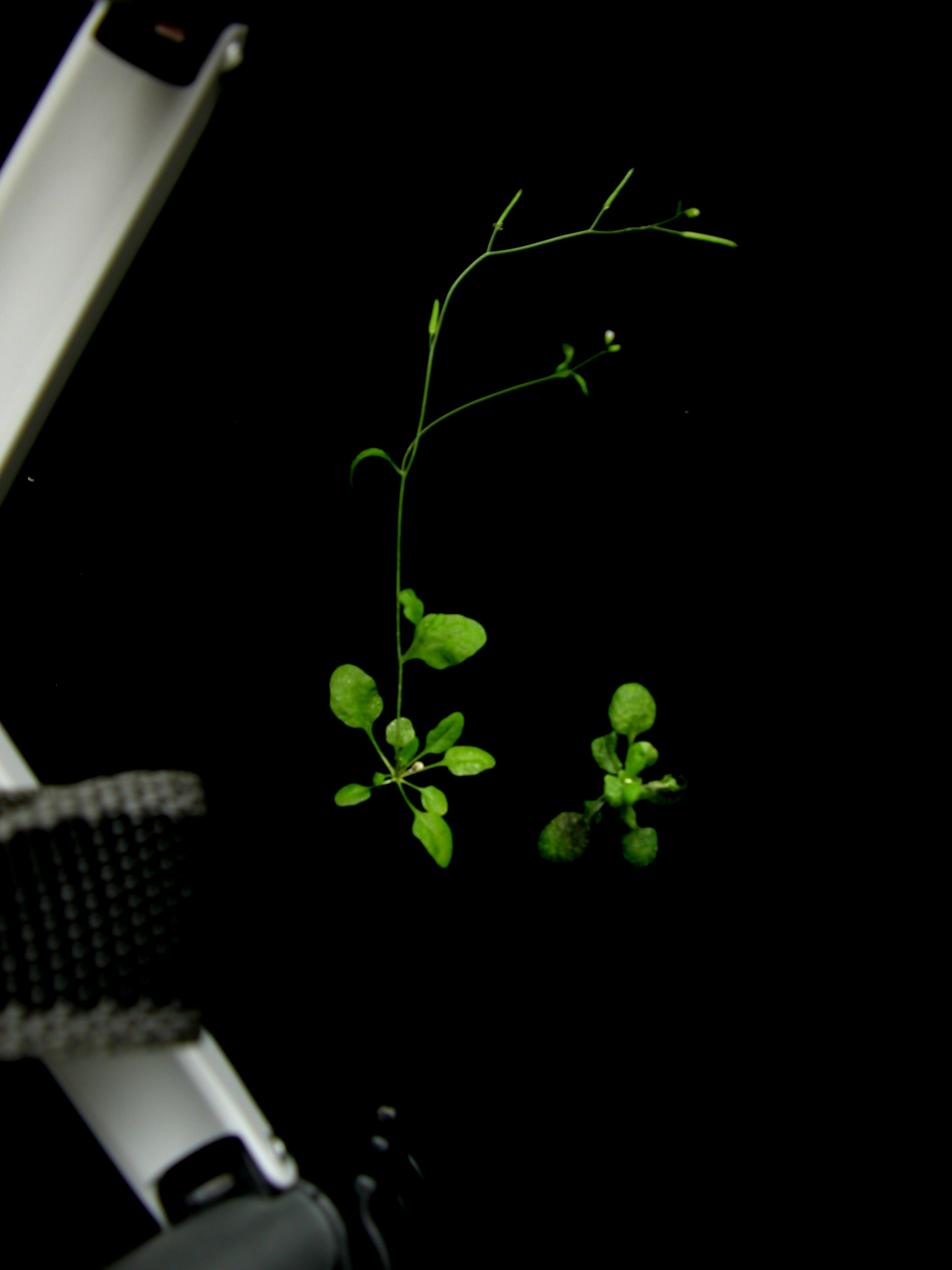

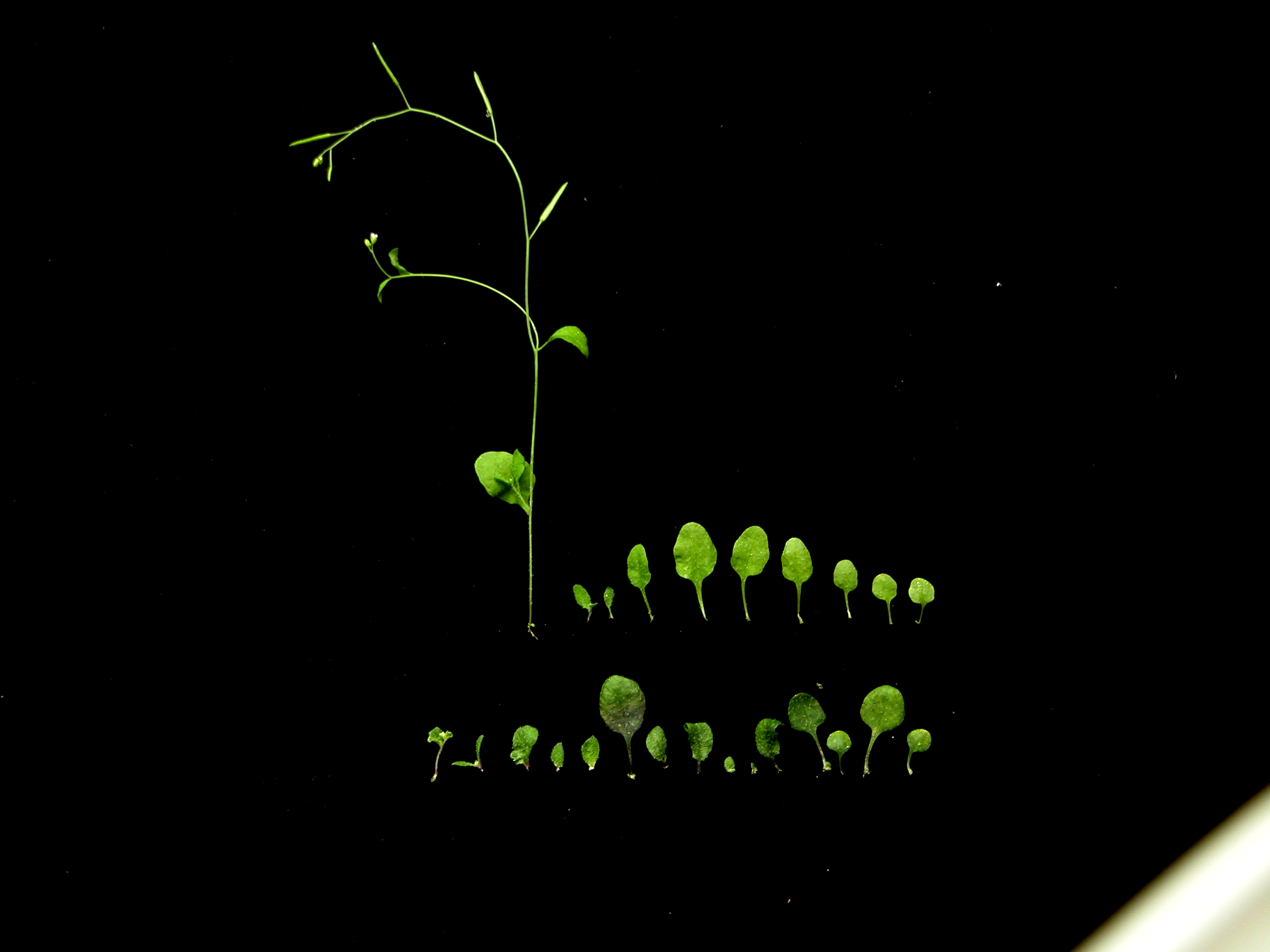

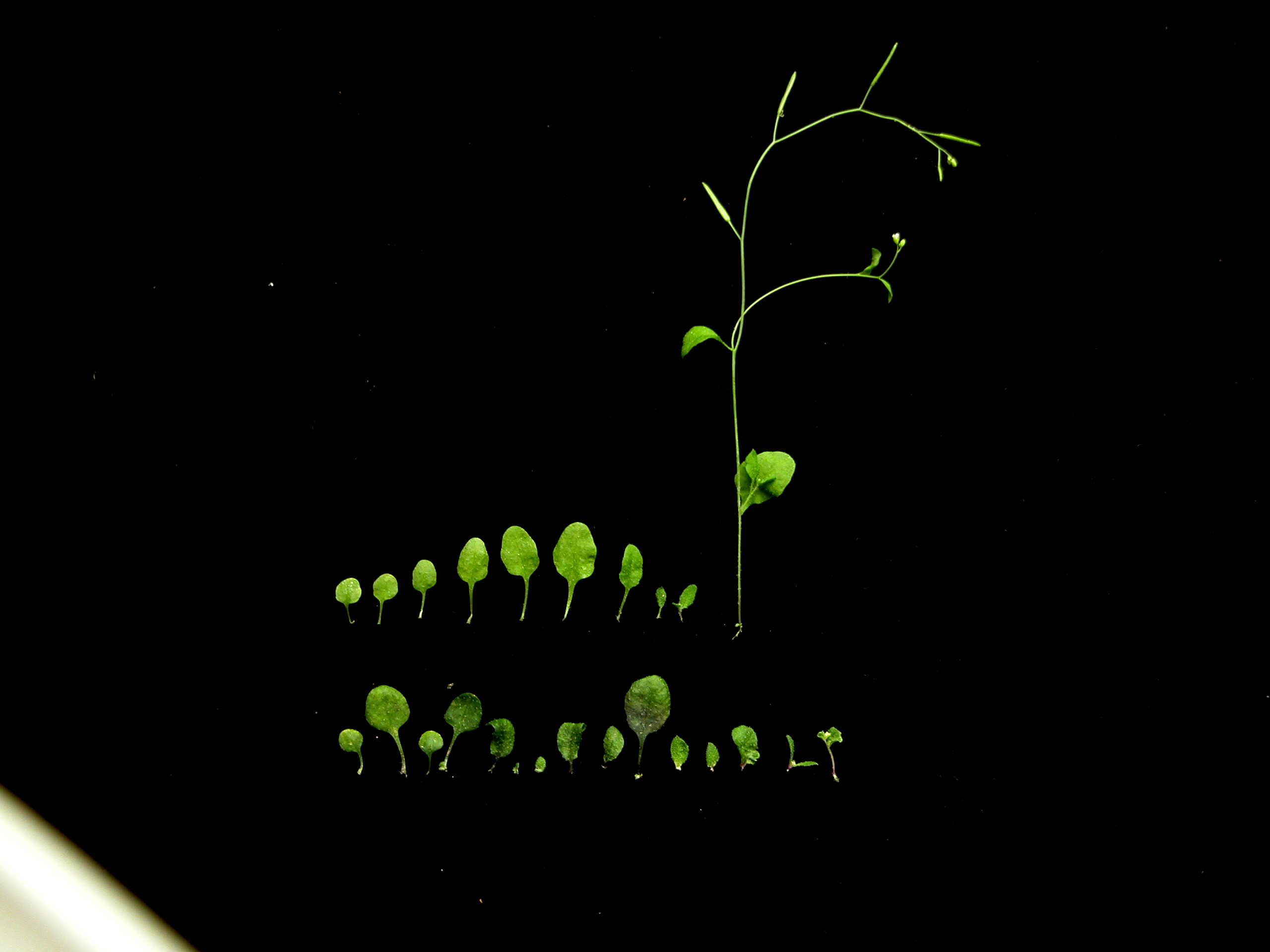

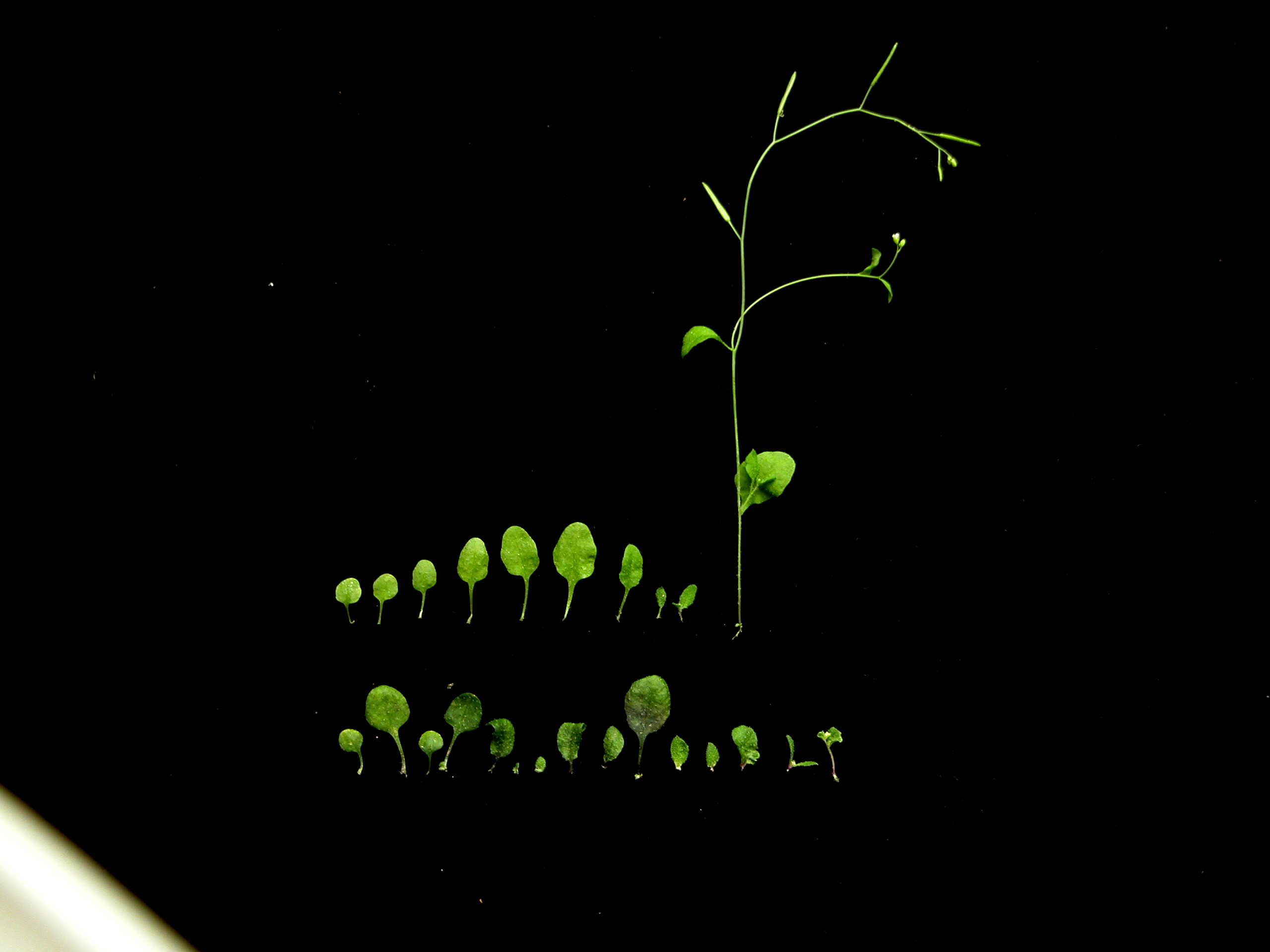

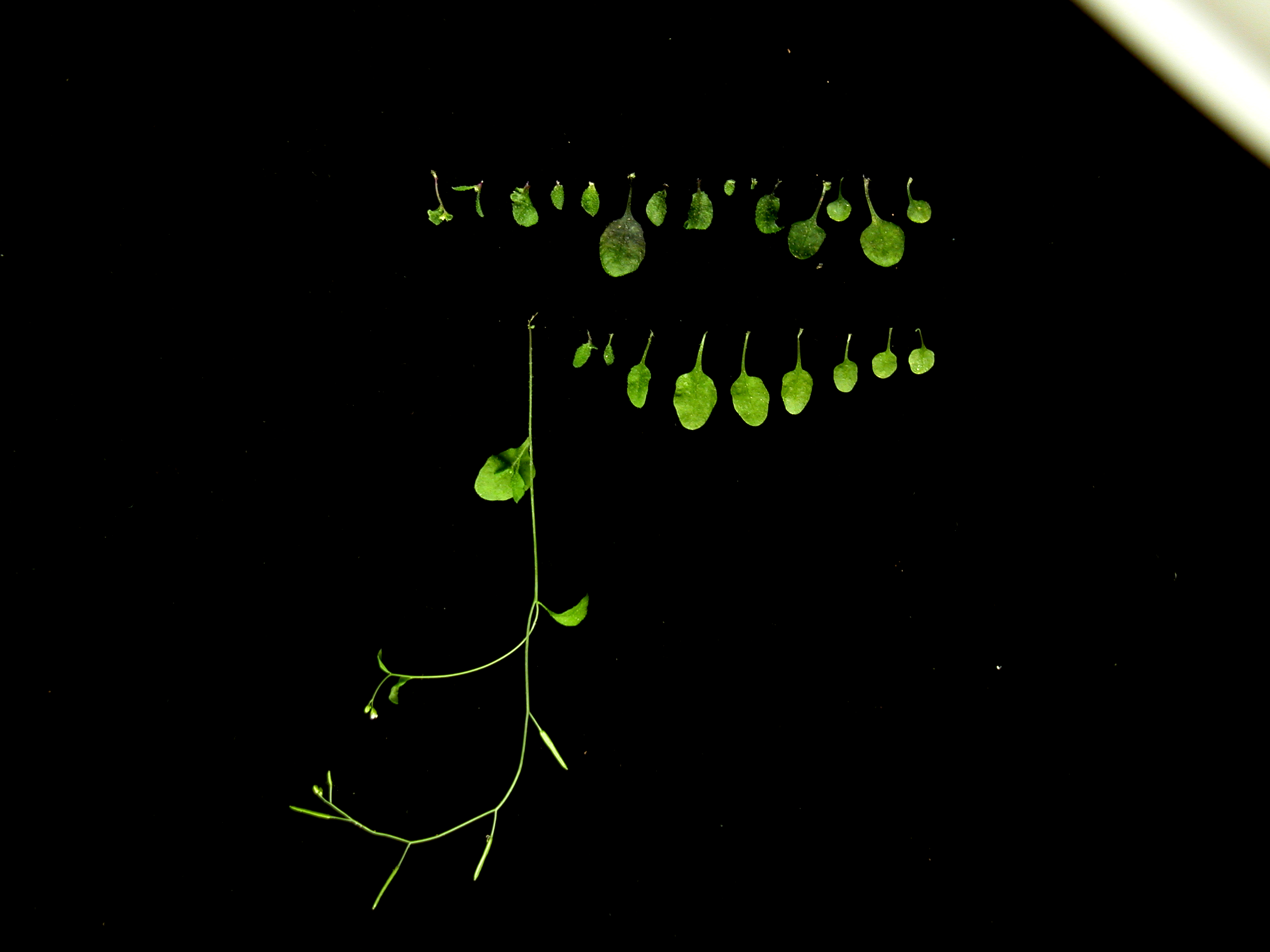

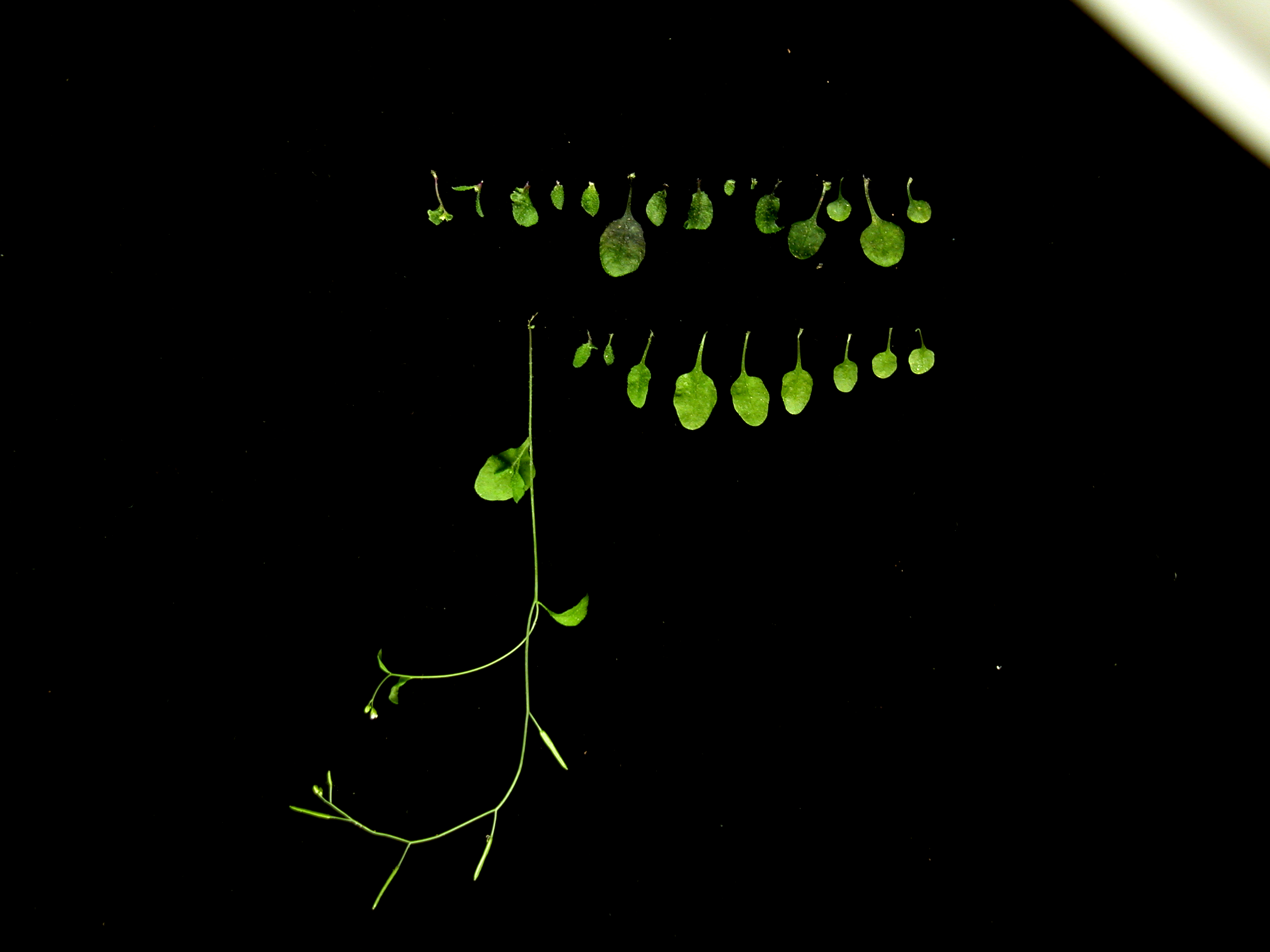


WT

OEMPT3

OEMPT3

WT


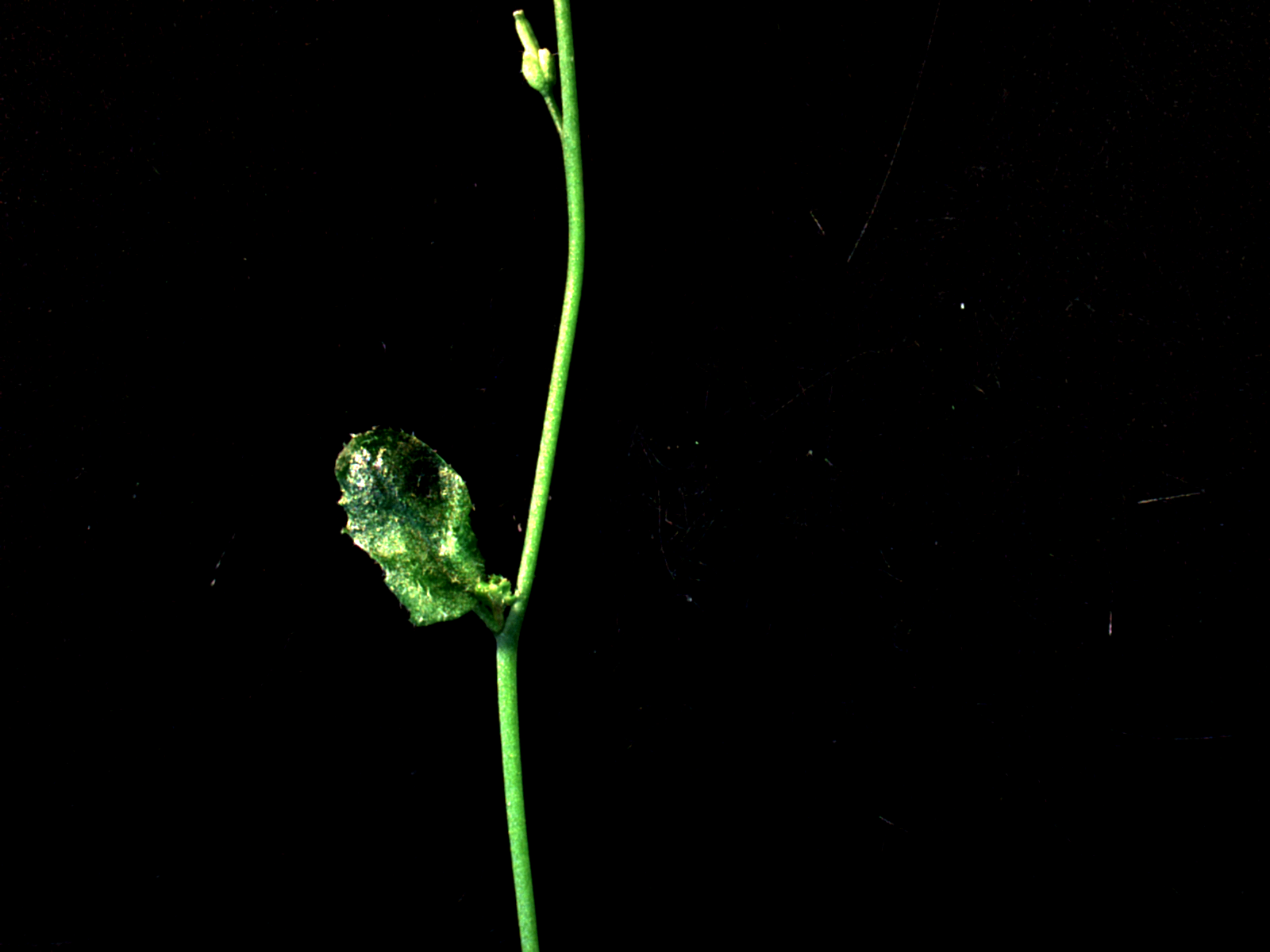

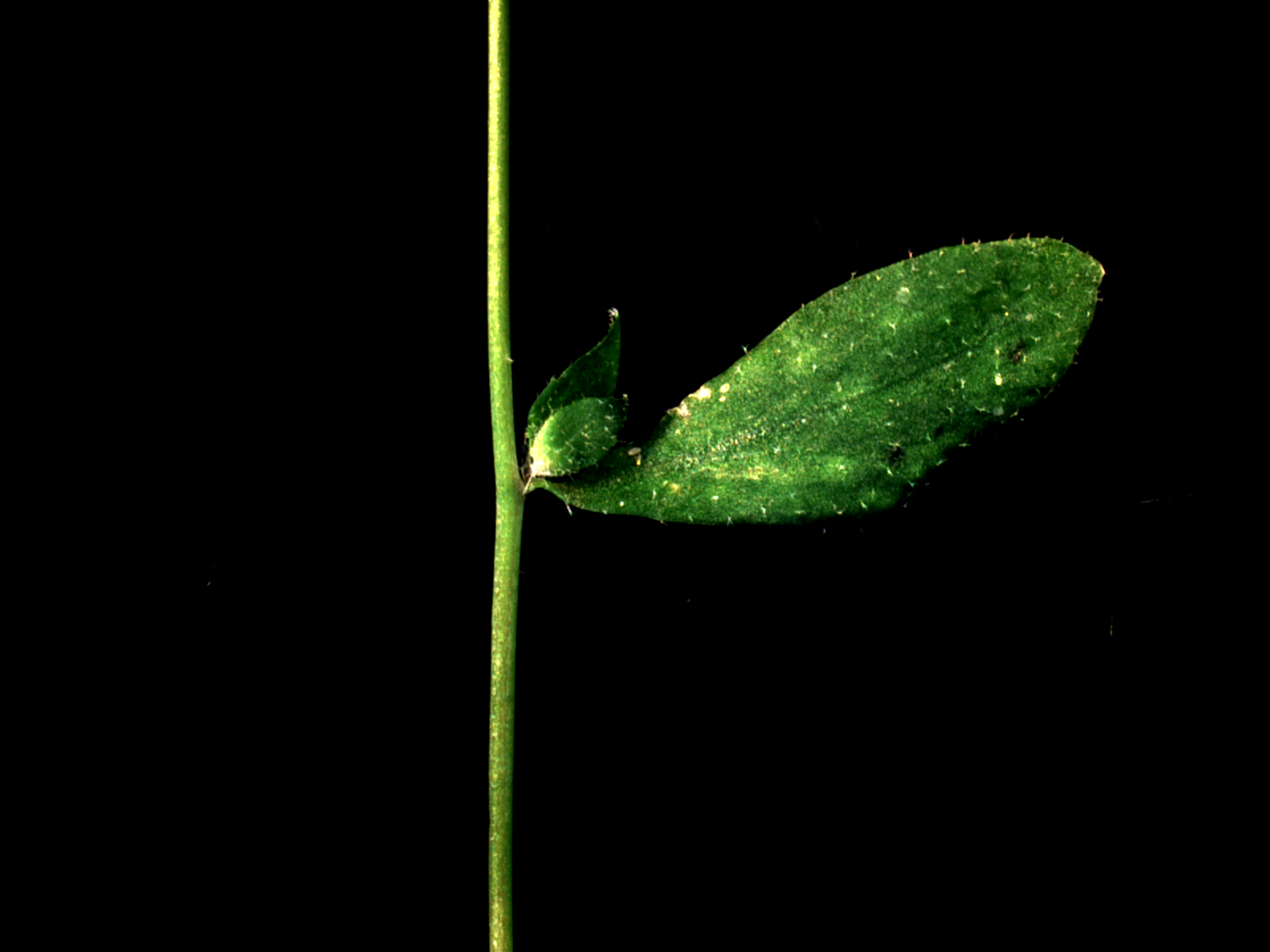


OEMPT3

WT

**C**

**B**

**A**

**A**

**B**

**Figure S6. Phenotypes of *AtMPT3* overexpressing plants.** (**A**) Late flowering phenotype of the six-week-old *AtMPT3* overexpressors compared to the wild-type plants was photographed. (**B**) Dark green distorted rosette leaves of the six-week-old *AtMPT3* overexpressors taken from the indicated genotypes were photographed. (**C**) Dark green distorted cauline leaves of the eight-week-old *AtMPT3* overexpressors taken from the indicated genotypes were photographed. OEMPTs, the *AtMPT* overexpressors.
