# Supplementary material for: The Mitochondrial Phosphate Transporters Modulate Plant Responses to Salt Stress via Affecting ATP and Gibberellin Metabolism in Arabidopsis thaliana
Source: PLoS One. 2012 Aug 24;7(8):e43530. doi: 10.1371/journal.pone.0043530 (PMC3427375; doi:10.1371/journal.pone.0043530)
Supplement: Figure S7 — Categories of biological processes for the AtMPT target genes. (DOC) [file pone.0043530.s007.doc]

**Figure S7**

**A**

GO: 0006811

Ion transport

GO: 0031668

Cellular response

to extrancellular stimulus

Up

GO: 0009991

Response to extracellular stimulus

GO: 0031667

Response to nutrient levels

GO: 0009636

Response to toxin

GO: 0009404

Toxin metabolic process

GO: 0019748

Secondary metabolic process

GO: 0044042

Glucan metabolic process

GO: 0006817

Phosphate transport

GO: 0010047

Fruit dehiscence

**B**

GO: 0009889

Regulation of biosynthetic process

GO: 0009719

Response to endogenous stimulus

GO: 0009733

Response to auxin stimulus

GO: 0010200

Response to chitin

GO: 0019219

Regulation of nucleobase, nucleoside, nucleotide and nucleic acid metabolic process

GO: 0045449

Regulation of transcription

Down

GO: 0009725

Response to hormone stimulus

GO: 0010033

Response to organic substance

GO: 0009743

Response to carbohydrate stimulus

**Figure S7.** **Categories of biological processes for the AtMPT target genes.** Categories of biological processes for the AtMPT target genes which were involved in the salt signaling were assigned based on the genome tool Genevestigator. These light- or dark-induced (**A**) and repressed (**B**) genes were classified into different biological processes by GOeast analysis. The darker the color is, the more significant the GO term is enriched in the dataset.
