# Supplementary material for: The Mitochondrial Phosphate Transporters Modulate Plant Responses to Salt Stress via Affecting ATP and Gibberellin Metabolism in Arabidopsis thaliana
Source: PLoS One. 2012 Aug 24;7(8):e43530. doi: 10.1371/journal.pone.0043530 (PMC3427375; doi:10.1371/journal.pone.0043530)
Supplement: Table S1 — The identity (%) between AtMPT proteins and the rice, yeast and bovine MPTs. (DOC) [file pone.0043530.s008.doc]

**Table S1. The identity (%) between AtMPT proteins and the rice, yeast and bovine MPTs.**

AtMPT1 AtMPT2 AtMPT3 OsMPT1 OsMPT2 OsMPT3 OsMPT4 OsMPT5 OsMPT6 Yeast MPT Bovine MPT-A Bovine MPT-B

AtMPT1 — 41.78 42.13 41.30 40.22 40.82 38.44 40.91 64.22 30.84 37.19 37.29

AtMPT2 — — 65.87 68.56 68.46 65.94 54.47 56.15 40.93 31.54 47.12 48.09

AtMPT3 — — — 74.74 74.02 75.73 51.32 60.00 41.60 31.59 47.34 48.14
