# Supplementary material for: The Mitochondrial Phosphate Transporters Modulate Plant Responses to Salt Stress via Affecting ATP and Gibberellin Metabolism in Arabidopsis thaliana
Source: PLoS One. 2012 Aug 24;7(8):e43530. doi: 10.1371/journal.pone.0043530 (PMC3427375; doi:10.1371/journal.pone.0043530)
Supplement: Table S2 — Names and annotations of target genes encoded the important signal transduction components. (DOC) [file pone.0043530.s009.doc]

Gene

Annotation

Gene

Annotation

AT4G25420 AtGA20ox1

AT5G51810 AtGA20ox2

AT5G07200 AtGA20ox3

AT1G60980 AtGA20ox4

AT1G44090 AtGA20ox5

AT1G15550 AtGA3ox1

AT1G80340 AtGA3ox2

AT1G80330 AtGA3ox3

AT4G21690 AtGA3ox4

AT1G78440 AtGA2ox1

AT1G30040 AtGA2ox2

AT2G34555 AtGA2ox3

AT1G47990 AtGA2ox4

AT1G02400 AtGA2ox6

AT1G50960 AtGA2ox7

AT4G21200 AtGA2ox8

AT1G06170 bHLH

AT2G27300 ANAC40

AT2G37030 ARF

AT1G66570 ATSUC7

AT5G49290 LRR

AT5G27810 AGL80

AT5G23470 NIF

AT3G51570 disease resistance protein

AT1G01520 MYB

AT5G24110 ATWRKY30

AT1G18710 ATMYB47

AT3G05650 ATRLP32

AT3G66656 AGL91

AT5G25160 ZFP3

AT2G45900 Exp-PT1

AT4G09600 GASA3

**Table S2. Names and annotations of target genes encoded the important signal transduction components.**
