# Supplementary material for: The Mitochondrial Phosphate Transporters Modulate Plant Responses to Salt Stress via Affecting ATP and Gibberellin Metabolism in Arabidopsis thaliana
Source: PLoS One. 2012 Aug 24;7(8):e43530. doi: 10.1371/journal.pone.0043530 (PMC3427375; doi:10.1371/journal.pone.0043530)
Supplement: Table S3 — Primers used for plasmid construction in this study. (DOC) [file pone.0043530.s010.doc]

**Table S3.Primers used for plasmid construction in this study**

| Gene name | AGI code | Orientation | Primer （5’-3’） |
| --- | --- | --- | --- |
| *AtMPT1* | At2g17270 | Forward | ATGACAAGAGTCAAGAGTAAACTAGAC |
|  |  | Reverse | TCACACTGATAATTTAGCTGCATCC |
| *AtMPT2* | At3g48850 | Forward | ATGTCTGACTCAAGCAGATCGC |
|  |  | Reverse | TTATGCACTTACAGATGGAGCAAG |
| *AtMPT3* | At5g14040 | Forward | ATGGAATCTCCGAAGAATTCTCTG |
|  |  | Reverse | TTAGGCTTTGGCTTCAGTAGCT |
| *AtMPT1-promoter* | At2g17270 | Forward | CTGCAGTCTTTGTGTGCTGTTTGATTATGTTA |
|  |  | Reverse | GGATCCCTTCTTACCAAAAATCGTTTCAAAA |
| *AtMPT2-promoter* | At3g48850 | Forward | CTGCAGCAGTGTGTTGGTGAAGGTACTTGTT |
|  |  | Reverse | GGATCCTTCGAGAGAGGAGATGAGAAATTC |
| *AtMPT3-promoter* | At5g14040 | Forward | CTGCAGTAGCTTCCTTAGACGGTTGACAC |
|  |  | Reverse | GGATCCGGCGGCGGCGGAGGAAGGA |
