# Supplementary material for: The Mitochondrial Phosphate Transporters Modulate Plant Responses to Salt Stress via Affecting ATP and Gibberellin Metabolism in Arabidopsis thaliana
Source: PLoS One. 2012 Aug 24;7(8):e43530. doi: 10.1371/journal.pone.0043530 (PMC3427375; doi:10.1371/journal.pone.0043530)
Supplement: Table S4 — Real-Time PCR primers used in this study. (DOC) [file pone.0043530.s011.doc]

**Table S4. Real-Time PCR primers used in this study**

| Gene name | AGI code | Orientation | Primer （5’-3’） |
| --- | --- | --- | --- |
| *AtGA20ox1* | [AT4g25420](http://www.arabidopsis.org/servlets/TairObject?id=26544&type=locus) | Forward | CGAACGATAGATACAAGAGC |
|  |  | Reverse | CGGACACAAGAAGAATGC |
| *AtGA20ox2* | AT5g51810 | Forward | GCCAGACGAAGAGAAACC |
|  |  | Reverse | ATTGACGACGAGGAAGAAG |
| *AtGA20ox3* | AT5g07200 | Forward | ACATCTCTAACCATACTTCATC |
|  |  | Reverse | ACTCTTGTATCTTCCATTCG |
| *AtGA20ox4* | At1g60980 | Forward | ACTGCGACCCAACCTCTC |
|  |  | Reverse | GCCAATGTTCACCACCAATG |
| *AtGA3ox1* | AT1g15550 | Forward | GCTCGTATCGCATCTTTC |
|  |  | Reverse | ATCGCAGTAGTTGAGGTG |
| *AtGA3ox2* | AT1g80340 | Forward | TGGTTCGCATTAGGTTCACTG |
|  |  | Reverse | TGGCTCTGTCTGGTTCTGG |
| *AtGA3ox4* | At1g80330 | Forward | GACTCCTCCCTACTTACCATTC |
|  |  | Reverse | GGCTCTACTCCAATCCATCTC |
| *AtGA2ox1* | AT1g78440 | Forward | CAAGAGCGTGAGGCATAGG |
|  |  | Reverse | GAAGGTCCAGCGAAGTAAATC |
| *AtGA2ox2* | AT1g30040 | Forward | ATCTCCTCCTCAATGCTAATCC |
|  |  | Reverse | TACTCCTCCACCGACTCAC |
| *AtGA2ox3* | AT2g34555 | Forward | GGAGCAAGAAGCCATCAAC |
|  |  | Reverse | TACTCAAGCCAGCCAAGG |
| *AtGA2ox4* | AT1g02400 | Forward | GTAAGGCATAGAGCATTGAC |
|  |  | Reverse | ACAACCGTGGCTGATTC |
| *AtGA2ox6* | AT1g02400 | Forward | TGAGCTTGAGTATCTTCTTC |
|  |  | Reverse | TAACCGTGCGTATGTAATC |
| *AtGA2ox7* | [AT1g50960](http://www.arabidopsis.org/servlets/TairObject?id=30916&type=locus) | Forward | TTCTTCGTATGTCCTTATCTC |
|  |  | Reverse | ATGTTCACTCTGCTCTTTG |
| *AtGA2ox8* | [AT4g21200](http://www.arabidopsis.org/servlets/TairObject?id=128364&type=locus) | Forward | TGGATGTGTTGGAGAAGATGAG |
|  |  | Reverse | CGGATAGAAGTGGCTGACG |
| *AtMPT1* | AT2g17270 | Forward | TGTCTTGCTGGCTATACTG |
|  |  | Reverse | CATTCTTGGCTTTGTTGTTG |
| *AtMPT2* | AT3g48850 | Forward | CGATTACGCCACTTGATG |
|  |  | Reverse | GCTCTTTGATTGTTGTCTTG |
| *AtMPT3* | AT5g14040 | Forward | AAAGAGCAAGGAGTCAAAGG |
|  |  | Reverse | CAGCAAGGTCAGAGTAAGTC |
